# Effects of different oxygen concentrations during intermittent hyperoxic training on endurance capacity in well‐trained male mice

**DOI:** 10.14814/phy2.71020

**Published:** 2026-07-18

**Authors:** Junichi Suzuki

**Affiliations:** ^1^ Laboratory of Exercise Physiology, Health and Sports Sciences, Course of Sports Education, Department of Education Hokkaido University of Education Iwamizawa Hokkaido Japan

**Keywords:** antioxidant enzyme, Bayesian data analysis, endurance capacity, hybrid exercise, intermittent hyperoxia

## Abstract

While training under intermittent hyperoxia (INT) enhances endurance capacity, the effects of specific oxygen concentrations remain unclear. This study compared 50% O_2_ (INT50) and 75% O_2_ (INT75) in well‐trained male mice, which had increased maximal work values by 7.7‐fold through a 7‐week voluntary running program, during a 4‐week treadmill program. Results showed that INT75, but not INT50, significantly improved endurance capacity compared to the normoxic‐trained (ET) group (Bayes factor, BF ≥3). The enhanced exercise capacity in the INT75 group was linked to higher levels of metabolic enzymes (citrate synthase, 3‐hydroxyacyl‐CoA‐dehydrogenase, carnitine palmitoyl transferase‐2, and phosphofructokinase) in the diaphragm and increased pyruvate dehydrogenase complex activity in the red gastrocnemius (Gr) and plantaris (PL) muscles (BF ≥3). Glutathione peroxidase‐1 protein levels in the PL muscle were significantly lower in INT75 (vs ET, 0.76‐fold, BF ≥3). Additionally, INT75 mice exhibited increased PGC1α levels in the left ventricle, despite decreased cytochrome‐c oxidase activity. In contrast, the INT50 group showed a shift toward fast‐twitch muscle fibers in the Gr. In conclusion, the ergogenic effects of intermittent hyperoxia depend on oxygen concentration. INT at 75% O_2_ significantly enhances endurance capacity in well‐trained mice by optimizing metabolic enzyme activity across skeletal and respiratory muscles.

## 1 INTRODUCTION

Endurance exercise capacity is a critical determinant of athletic performance and a key indicator of metabolic properties. While traditional aerobic training, characterized by prolonged sessions at submaximal intensities, reliably improves cardiovascular function and muscular endurance, athletes often reach a physiological plateau where further gains become difficult to achieve. To overcome these limits, researchers have explored various supplemental strategies, including manipulating inspired oxygen fractions, such as hypoxia, hyperbaric hyperoxia, or normobaric hyperoxia.

Hyperoxic training, or training while breathing air with an oxygen concentration greater than 21%, has emerged as a potential ergogenic aid (Ulrich et al., 2017). The theoretical basis for hyperoxia lies in its ability to increase arterial partial pressure of oxygen (PO_2_) through oxygen saturation of hemoglobin and dissolved oxygen in the plasma, thereby enhancing oxygen delivery to the working muscles. Inspired 60% O_2_ during exercise increased arterial PO_2_ levels to approximately 300 Torr (Mourtzakis et al., 2004). The increased oxygen availability, facilitated by enhancing the PO_2_ gradient between microvessels and mitochondria, likely allows for higher training intensities, altering metabolic demands and leading to superior adaptations compared to normoxic training.

Exercise training under continuous hyperoxia has not shown beneficial results for improving exercise capacity or muscle metabolic properties (Kon et al., 2019, 2020; Perry et al., 2005; Suzuki, 2024, 2025). It has been proposed that hyperoxic exposure followed by normoxia is interpreted as a hypoxic event at the cellular level, a phenomenon known as the hyperoxic‐hypoxic paradox (Salvagno et al., 2023). Thus, repeated hyperoxic and normoxic exposure may stabilize hypoxia‐inducible factor (HIF) 1α (Balestra et al., 2006; Cimino et al., 2012). In humans, breathing 100% oxygen for 2 h, followed by 36 h of room air, resulted in a 60% increase in serum erythropoietin (EPO) levels (Balestra et al., 2006). Acute intermittent hyperoxic exposure (three cycles of 21% O_2_ for 10 min and 30% O_2_ for 15 min), followed by 3 h of rest in room air, upregulated HIF1α target genes (phosphofructokinase [PFK] and vascular endothelial growth factor [VEGF]), as well as genes known to promote muscle metabolic properties (mitochondrial transcription factor A, peroxisome proliferator‐activated receptors‐α and ‐γ) (Suzuki, 2024).

Recent evidence suggests that intermittent hyperoxia, characterized by repeating short‐duration exposure to 30% O_2_ during exercise sessions, may trigger distinct molecular and enzymatic changes (Suzuki, 2024, 2025). Specifically, alterations in mitochondrial enzyme activity, such as citrate synthase (CS) and cytochrome‐c oxidase (COX), as well as shifts in fuel‐providing enzymes like pyruvate dehydrogenase complex (PDHc) and hydroxyacyl‐CoA dehydrogenase (HAD), are believed to mediate these improvements.

Despite these theoretical benefits, the dose–response relationship between oxygen concentration and endurance adaptation is not fully understood, particularly in already “well‐trained” subjects who may have reduced sensitivity to standard training stimuli. Most studies have focused on sedentary or moderately active models, leaving a knowledge gap regarding the “additive effects” of hyperoxia on an established high‐exercise capacity baseline. In college male athletes, high‐intensity interval training under hyperoxia (60% O_2_) did not improve maximal oxygen uptake values (Kon et al., 2019, 2020). Thus, any ergogenic effect of hyperoxic training has yet to be observed in well‐trained athletes.

In streptozotocin (STZ)‐induced diabetic rats, hyperoxic exposure (50% O_2_, 1 h per day for 4 weeks) significantly decreased the proportion of type I fibers and increased that of type II fibers in the extensor digitorum longus (EDL) muscle (Sugimoto et al., 2025). However, these fiber type shifts were not observed in the group exposed to 40% O_2_. Fiber‐type transitions have been shown to directly influence exercise capacity (Wilson et al., 2012). Thus, it was hypothesized that the improvement in exercise capacity induced by the INT protocol would be dose‐dependent on the oxygen concentration utilized.

In the present study, experiments were designed to investigate whether the beneficial effects of exercise training under intermittent hyperoxia (50% or 75% O_2_) depend on oxygen concentration. To clarify this, the author analyzed adaptive changes in muscle metabolic features, muscle capillary and fiber type properties, and antioxidant protein levels in the hind leg muscles, diaphragm, and heart.

## 2 MATERIALS AND METHODS

### 2.1 Ethical approval

All procedures were approved by the Animal Care and Use Committee of Hokkaido University of Education (approved on 2025/3/31) and conducted in accordance with the “Guiding Principles for the Care and Use of Animals in the Field of Physiological Sciences” of the Physiological Society of Japan.

### 2.2 Animals

**FIGURE 1:**
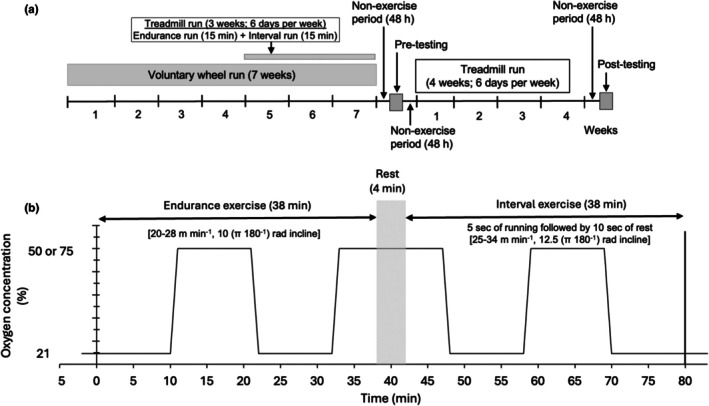
Experimental design. (a) Whole experimental protocols; (b) Daily experimental protocols.

The present experimental design was summarized in Figure 1. Forty male MCH(ICR)/jcl mice, aged 3 weeks, were purchased from Clea Japan (Tokyo, Japan). They were housed under controlled conditions, with a temperature of 24°C ± 1°C and a relative humidity of approximately 50%. Lighting was automatically controlled from 7:00 to 19:00. All mice were given commercial laboratory chow (solid CE‐2; Clea Japan) and tap water ad libitum. After the mice had been fed for 2 weeks and allowed to adapt to the new environment, they were randomly assigned to a sedentary control group (SED, *n* = 10) or training group (*n* = 30).

#### 2.2.1 Voluntary wheel run

Mice in the training group were individually housed in a cage with a wheel activity device (13 cm in diameter) for 7 weeks. Wheel activity (distance and running time) was monitored and recorded using digital bike computers (CC‐VL820; Cateye, Osaka Japan). Running distance per day during voluntary wheel training was shown in Figure S1. To familiarize mice with a treadmill device, mice in the SED group were subjected to walking once a week on a treadmill (KN‐73, Natsume, Tokyo, Japan) for 3 min per day at 10–15 m min^−1^ with a 5 (π 180^−1^) rad incline. Mice in the training group were subjected to the familiarization until the fourth week, and thereby to treadmill exercise regimen during the 5th through 7th weeks of voluntary running. The regimen consisted of 15 min of endurance running, followed by a 2‐min rest, and then an interval run (5 s of running followed by 10 s of rest) for an additional 15 min. In the 5th week, the treadmill had a 7.5 (π 180^−1^) rad incline, which increased to 10 (π 180^−1^) rad from the 6th week onward. The speed for the endurance run was set at 15 m min^−1^ during the 5th and 6th weeks and increased to 20 m min^−1^ in the 7th week. For the interval run, the speed was 20 m min^−1^ in the 5th week and 25 m min^−1^ from the 6th week onward.

#### 2.2.2 Maximal exercise capacity test (pre‐testing)

Following the voluntary training, the mice were given a 48‐h non‐exercise period before the maximal exercise capacity test. This test was conducted using a graded ramp running protocol on the treadmill, as shown in Figure S2. Total work (J) was calculated by multiplying body weight (kg), speed (m s^−2^), time (sec), slope (%), and 9.8 (m s^−2^). Exhaustion was defined as the condition in which the mouse remained on the metal grid at the rear of the treadmill for more than 5 s (without electrical shock), despite gentle external stimulation applied to the tail with a bamboo stick (0.8 mm in diameter).

#### 2.2.3 Hybrid exercise training

**FIGURE 2:**
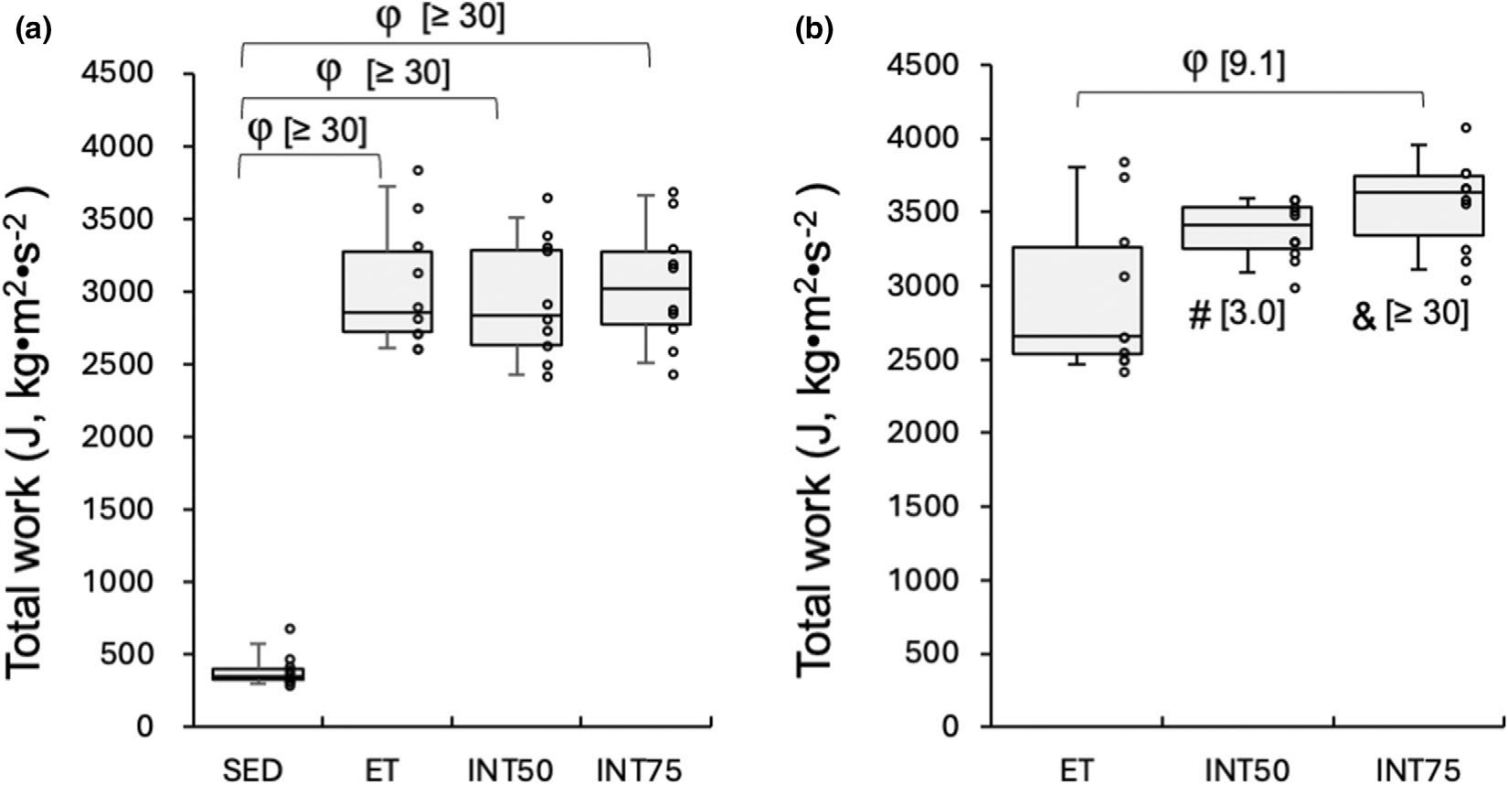
Results of endurance exercise capacity test. Total work values of the endurance capacity test (a) before and (b) after 4 weeks of treadmill exercise training. Values are expressed as box and whisker plots with 5th, 25th, 50th, 75th, and 95th percentile. Dots in the figure indicate data for each mouse. #, significantly different (BF ≥15) from pre‐treadmill training values of each group shown in the panel (a). Bayes factors are in parentheses. φ, The 95% confidence interval did not contain the mean value of target group for comparison.

After the test, mice in the training group underwent exercise training for 4 weeks (Figure 1a). Voluntary trained mice were divided into a normoxic exercise‐trained group (ET, *n* = 10) and an exercise‐trained under intermittent hyperoxia (50% O_2_ [INT50, *n* = 10] or 75% O_2_ [INT75, *n* = 10]) groups to match the mean and standard deviation (SD) values for total work (Mean ± SD, ET, 3030.3 ± 429.2; INT50, 2943.5 ± 419.3; INT75, 3048.7 ± 418.4; Figure 2a). All mice in the trained groups successfully completed the exercise training and their results were included in this study.

The mice in the training groups underwent hybrid exercise training, as shown in Figure 1b, for 4 weeks, 6 days a week. The hybrid exercise lasted for 80 min and consisted of an endurance run for 38 min with a 10 (π 180^−1^) rad incline, followed by 4 min of rest on the treadmill, and interval exercise for 38 min, with a 12.5 (π 180^−1^) rad incline. Instead of using electrical shock, the mice were motivated to run by gently touching their tail or planta pedis with a conventional test tube brush made of soft porcine bristles if they remained on a metal grid for more than 3 s.

On the first day, the mice ran at 20 m min^−1^ for endurance exercise. The speed was gradually increased to 26 m min^−1^ and 28 m min^−1^ on the 7th and 14th days of the training period, respectively. For interval exercise (5 s of running followed by 10 s of rest), the mice ran at 25 m min^−1^ on the first day. The speed was gradually increased to 27.5, 30, 32, and 34 m min^−1^ on the 2nd, 4th, 7th, and 14th days of the training period, respectively. Each exercise intervention took place between 5 am and 9 am, and the order of the interventions was randomized daily.

#### 2.2.4 Exercise with intermittent hyperoxia

The INT group exercised under intermittent hyperoxia, consisting of three cycles of 50% or 75% O_2_ for 15 min, followed by room air for 10 min, as illustrated in Figure 1b, using the treadmill. During the hyperoxia sessions, the treadmill runway was covered with a translucent plastic film to create a runway chamber, measuring 1.10 m in length, 0.78 m in width, and 0.3 m in height. To maintain CO_2_ concentrations below 1000 ppm, air in the chamber was circulated through a CO_2_ absorbent (Litholyme; Allied Healthcare Products, St. Louis, MO, USA).

When the O_2_ concentration was increased from 21% to 50% or 75%, 100% O_2_ was introduced into an air‐mixture box (length 0.32 m, width 0.17 m, and height 0.10 m) installed within the runway chamber. Inside the air‐mixture box, 100% O_2_ was mixed with circulated air (180 L min^−1^, using three closed air circulating pumps, VP6035S, Techno Takatsuki, Osaka, Japan) for CO_2_ absorption, as previously described (Suzuki, 2024). The mixed air was then introduced into the runway chamber via a commercial circulator, ensuring that 100% O_2_ was not directly introduced into the chamber.

The O_2_ concentrations in both the air‐mixture box and the runway chamber were monitored using two sets of oxygen analyzers (G‐1690 and GOX‐100; Greisinger, Germany). When the O_2_ concentration was reduced from 50% or 75% to 21%, room air was introduced into the chamber by opening the runway chamber while simultaneously removing air from the chamber using a combined vacuum cleaner (MCU21, Panasonic, Osaka, Japan). Additionally, room air in the laboratory was continuously ventilated through a draft chamber (DF17C, Dalton, Tokyo, Japan).

#### 2.2.5 Post‐testing and sampling

The maximal endurance capacity test was performed 48 h after the last run, as described above. Forty‐eight hours after the exercise capacity test, the mice were anesthetized with 3% sevoflurane (193‐17791; Fujifilm‐Wako, Osaka, Japan) inhalation using an anesthetizer (MKA100W, Muromachi Kikai, Tokyo, Japan), and the adequacy of anesthesia was validated using a toe pinch response. The soleus (SOL), plantaris (PL), and gastrocnemius muscles were excised, and the deep red region (Gr) of the gastrocnemius was separated from the superficial white region (Gw). The diaphragm (DIA) was also excised. All samples were frozen in liquid nitrogen for biochemical analyses. The mice were killed by excision of the heart. After excision, the whole heart and left ventricle (LV) were weighed. All tissue samples were stored at −80°C until further analyses.

### 2.3 Sample preparation for biochemical analyses

A cytoplasmic fraction of protein was obtained as previously reported (Suzuki, 2022). Frozen tissue powder was obtained using a frozen sample crusher (SK mill; Tokken, Chiba, Japan) and homogenized with ice‐cold medium (10 mM HEPES buffer, pH 7.4; 1% NP‐40 [Fujifilm‐Wako]; 11.5% [w/v] sucrose; and 5% [v/v] protease inhibitor cocktail [P2714; Sigma‐Aldrich, St. Louis, MD, USA]) in an ultrasonic bath (43 kHz, 50 W) at 4°C for 5 min. It was then gently rotated at 4°C for 10 min. After centrifugation at 18,000*g* and at 4°C for 10 min, the supernatant, cytoplasmic fraction, was collected and stored at −80°C. Total protein concentrations were measured using the Bradford assay (0.01% [w/v] CBB G‐250 [B3193, Tokyo Chemical Industry, Tokyo, Japan], 5% [v/v] ethanol [054‐07225, Fujifilm‐Wako, Osaka, Japan], 8.5% [v/v] phosphoric acid [164‐02176, Fujifilm‐Wako]) with bovine serum albumin (21011, iNtRON Biotechnology, Korea) as a standard.

### 2.4 Western blot analyses

A sample containing 50 μg of cytoplasmic protein was heated at 99°C for 5 min with a Laemmli sample buffer (161‐0747, Bio‐Rad Laboratories, Hercules, CA, USA). Then the sample was separated on 12% polyacrylamide gels (TGX StainFree FastCast gel, 1610185, Bio‐Rad) using SDS/PAGE. The gels were exposed to ultra‐violet (UV) light for 1 min, and total protein patterns were visualized using the ChemiDoc MP Imager (Bio‐Rad). The stain‐free gel contains a trihalo compound that reacts with proteins during separation, making them detectable with UV exposure (Gilda & Gomes, 2013). The gels were then electrophoretically transferred to a polyvinylidene fluoride membrane (Hybond LFP, 10600022, Amersham, Cytiva, UK). The blot was blocked with 5% nonfat dry milk (190‐12865; Fujifilm‐Wako) in 0.1 M phosphate‐buffered saline (PBS) with 0.05% Tween20 for 1 h. Next, the blot was exposed to a specific primary antibody against peroxisome proliferator‐activated receptor gamma coactivator 1‐alpha (PGC1α, 1:1000, sc‐518025, Santa Cruz Biotechnology, Dallas, TE, USA), catalase (CAT, 1:1500, sc‐271803, Santa Cruz), superoxide dismutase‐1 (SOD1, 1:2000, sc‐101523, Santa Cruz), or glutathione peroxidase (GPx1/2, 1:750, sc‐133160, Santa Cruz) diluted in blocking buffer for 1 h. Complete images of all protein bands and the specific bands of the target protein detected after immunostaining are provided as Figures S5–S16. Based on the molecular weight of the detected bands (Figures S11–S13), GPX1 protein was identified in the present procedures. After incubating the blot with a HRP‐labeled mouse IgGκ light chain binding protein (1:6000, sc‐516102, Santa Cruz), it was reacted with Clarity Western ECL substrate (1705060, Bio‐Rad), Clarity Max Western ECL substrate (1705062, Bio‐Rad), or a mixture of both. The target proteins were detected with the ChemiDoc MP (Bio‐Rad). The densities of specific bands were quantified using Image Lab software (Bio‐Rad) and normalized to the densities of all protein bands in each lane on the membrane (Suzuki, 2021). This normalization procedure was confirmed to be superior to using β‐actin as a loading control (Gilda & Gomes, 2013). Subsequently, the normalized densities of the bands were further adjusted to the same sample run on every gel and transferred to every membrane, as previously reported (Suzuki, 2021).

### 2.5 Biochemical analyses of enzyme activity

The activity of 3‐hydroxyacyl‐CoA‐dehydrogenase (HAD) was assayed using the method described by Bass et al. (1969). Assay for cytochrome c oxidase (COX) activities were prepared according to the method of Sherratt et al. (1988). The activity of citrate synthase (CS) was assayed following the methods of Srere (1969). Pyruvate dehydrogenase complex (PDHc) activity was assayed according to the method of Ke et al. (2014). The activity of carnitine palmitoyl transferase (CPT) 2 was assayed as previously reported (Suzuki, 2021). Specific lactate dehydrogenase activities, pyruvate‐to‐lactate (LDH‐PL) or lactate‐to‐pyruvate (LDH‐LP) conversions, were determined following the protocol of Howell et al. (1979) with some modifications as reported previously (Suzuki, 2022). All measurements were performed in duplicate per sample at 25°C using a spectrophotometer (U‐2001; Hitachi Co., Tokyo, Japan). Enzyme activities are reported as micromoles per hour per milligram of protein.

### 2.6 Histological analyses

Representative immunofluorescent images for muscle fiber phenotypes and capillary profiles were shown in Figures S3 and S4, respectively. Histochemical examinations of capillary profiles and muscle fiber phenotypes were conducted as previously reported with slight modifications (Suzuki, 2019). Briefly, four 12‐micrometer‐thick cross‐sections for each sample were obtained from the mid‐belly of calf muscles using a cryotome (MRS; Nihon Kouki Seisakusyo, Nagano, Japan) in a −20°C freezer. These sections were air‐dried, fixed with 100% ethanol at 4°C for 15 min, incubated in 0.1 M phosphate‐buffered saline (PBS) with 0.1% Triton X‐100, and washed in PBS. Sections were then blocked with 3% bovine serum albumin (010‐25783, Fujifilm‐Wako) at room temperature for 30 min, washed in PBS for 5 min, and incubated at 4°C overnight with a mixture of an anti‐type I myosin heavy chain (MHC) antibody (BA‐F8; mouse IgG2b, 1:80), and anti‐type IIA MHC antibody (SC‐71, mouse IgG1, 1:80) diluted with PBS. Sections were then reacted with Alexa Fluor 350‐labeled anti‐mouse IgG2b (1:500, A21140), Alexa Fluor 647‐labeled anti‐mouse IgG1 (1:500, A21240), and fluorescein‐labeled Griffonia simplicifolia lectin (GSL‐I) (1:100, [FL 1101; Vector Laboratories, Burlingame, CA, USA]) diluted with PBS at room temperature for 2 h. Sections were coverslipped with Fluoromount/Plus (K048; Diagnostic BioSystems, Pleasanton, CA, USA). Primary and secondary antibodies were purchased from the Developmental Studies Hybridoma Bank (University of Iowa) and Thermo Fisher Scientific (Tokyo, Japan), respectively. Fluorescent images of the incubated sections were observed using a microscope (Axio Observer; Carl Zeiss Japan, Tokyo, Japan) using an objective lens (Objective EC Plan‐Neofluar 20×/0.50 M27, #420350‐9900‐000, Carl Zeiss). All images were captured using Zen pro 2012 software (Carl Zeiss). For each muscle (SOL and PL) or muscle portion (GrL, Grm, and Gw), microscopic images of 0.57 mm^2^ were obtained and analyzed. Muscle fiber phenotypes were classified as type I (blue), type IIA (red and purple), and type II (including IIAX and IIB+IIX, faint red and unstained, Figure S3). Fluorescent images were obtained from SOL, PL, the lateral (GrL) and medial (GrM) portions of Gr, and Gw. The negative control without primary antibodies was confirmed to show no fluorescent signal.

Non‐overlapping microscopic fields were selected at random from each tissue sample. The observer was blinded to the source (groups) of each slide during the measurements using a random number table.

### 2.7 Statistical analyses

#### 2.7.1 Procedures for significant testing

**FIGURE 3:**
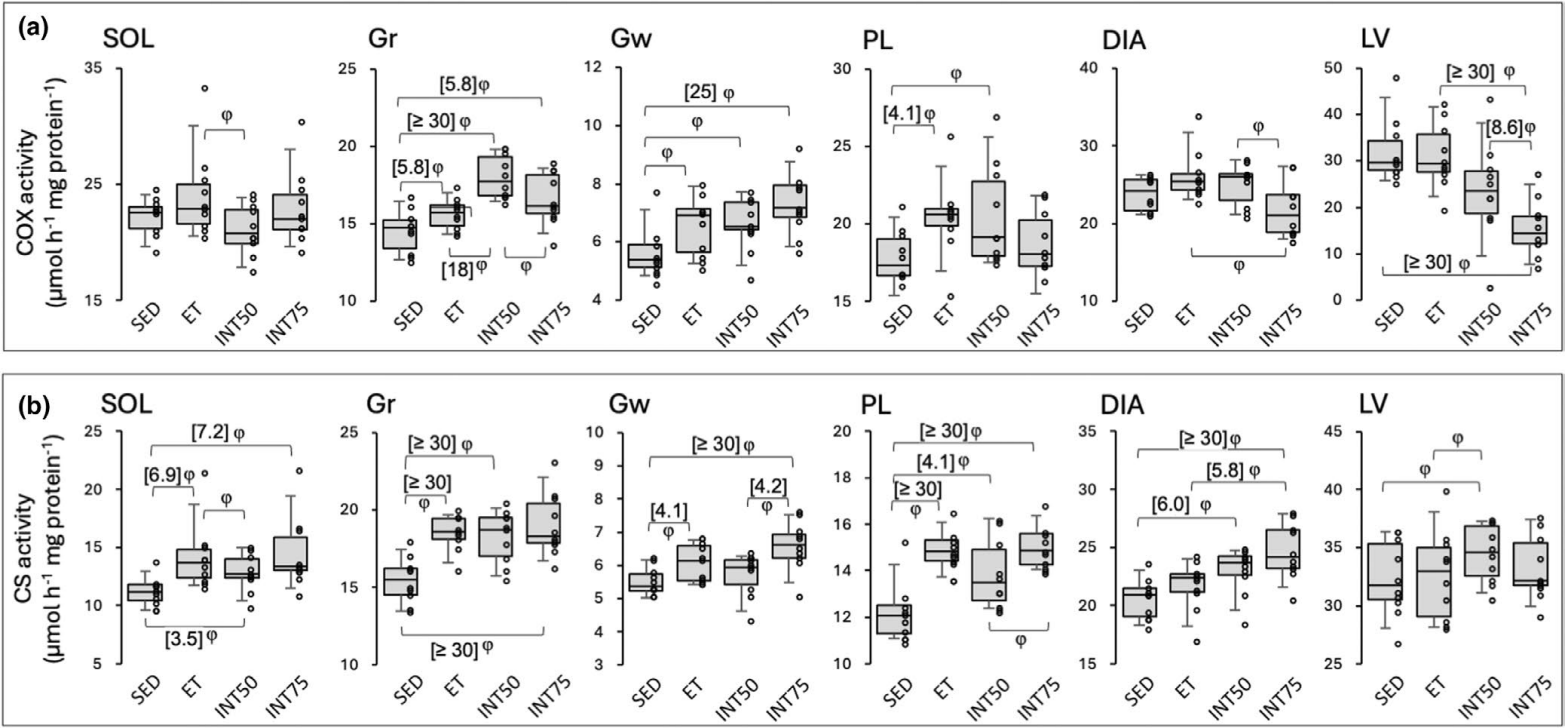
Enzyme activity values for COX (a) and CS (b) in Experiment‐2. Values are expressed as box and whisker plots with 5th, 25th, 50th, 75th, and 95th percentile. Dots are individual data points. Bayes factors are in parentheses. φ, The 95% confidence interval did not contain the mean value of target group for comparison.

**FIGURE 4:**
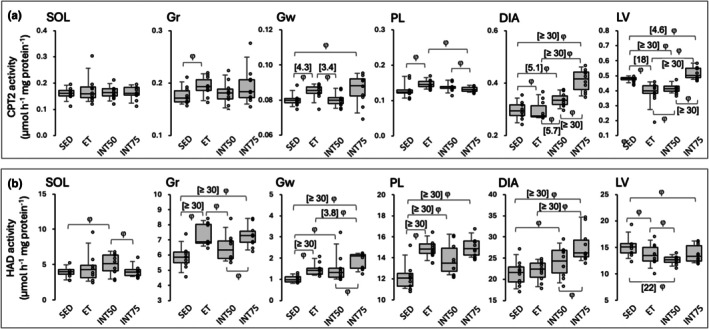
Enzyme activity values for CPT2 (a) and HAD (b) in Experiment‐2. Values are expressed as box and whisker plots with 5th, 25th, 50th, 75th, and 95th percentile. Dots are individual data points. Bayes factors are in parentheses. φ, The 95% confidence interval did not contain the mean value of target group for comparison.

**FIGURE 5:**
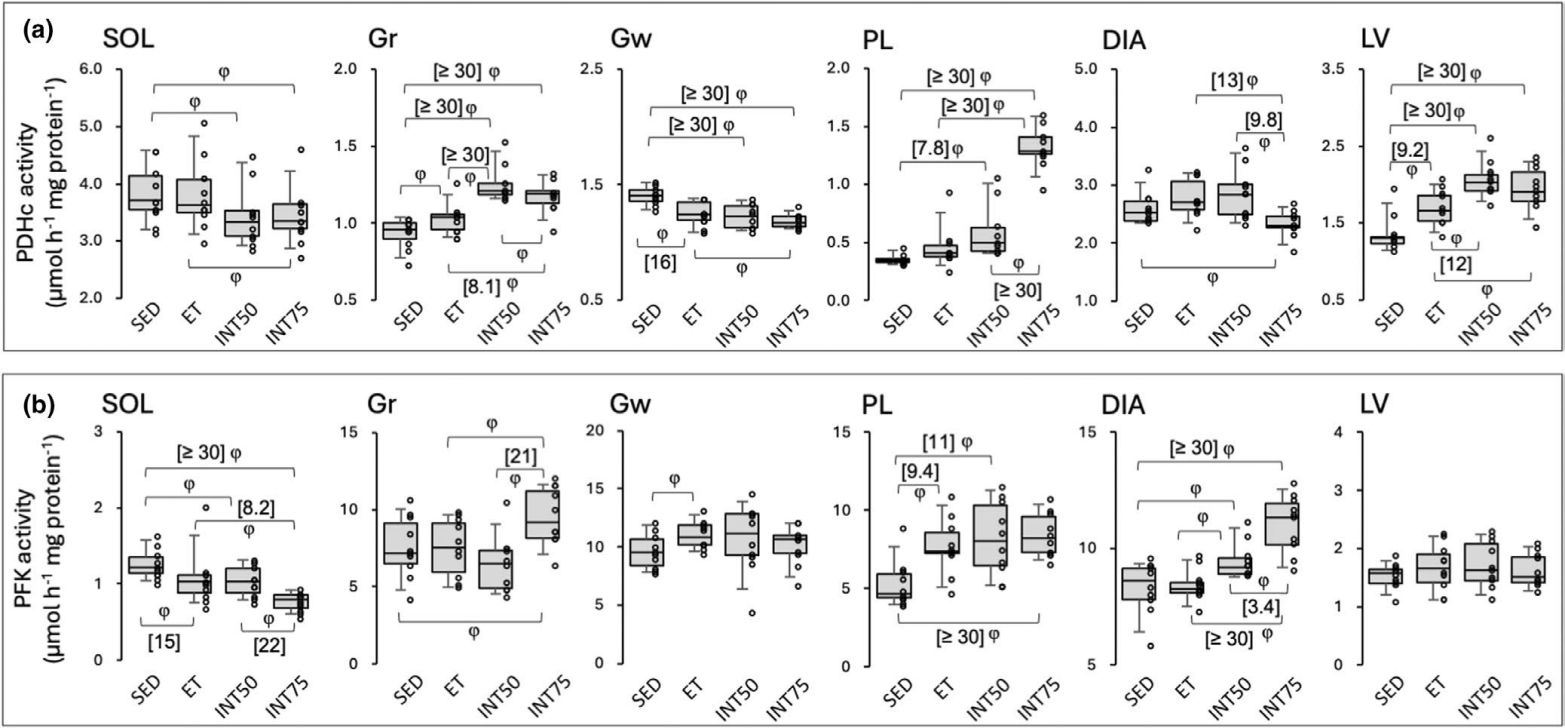
Enzyme activity values for PDHc (a) and PFK (b) in Experiment‐2. Values are expressed as box and whisker plots with 5th, 25th, 50th, 75th, and 95th percentile. Dots are individual data points. Bayes factors are in parentheses. φ, The 95% confidence interval did not contain the mean value of target group for comparison.

**FIGURE 6:**
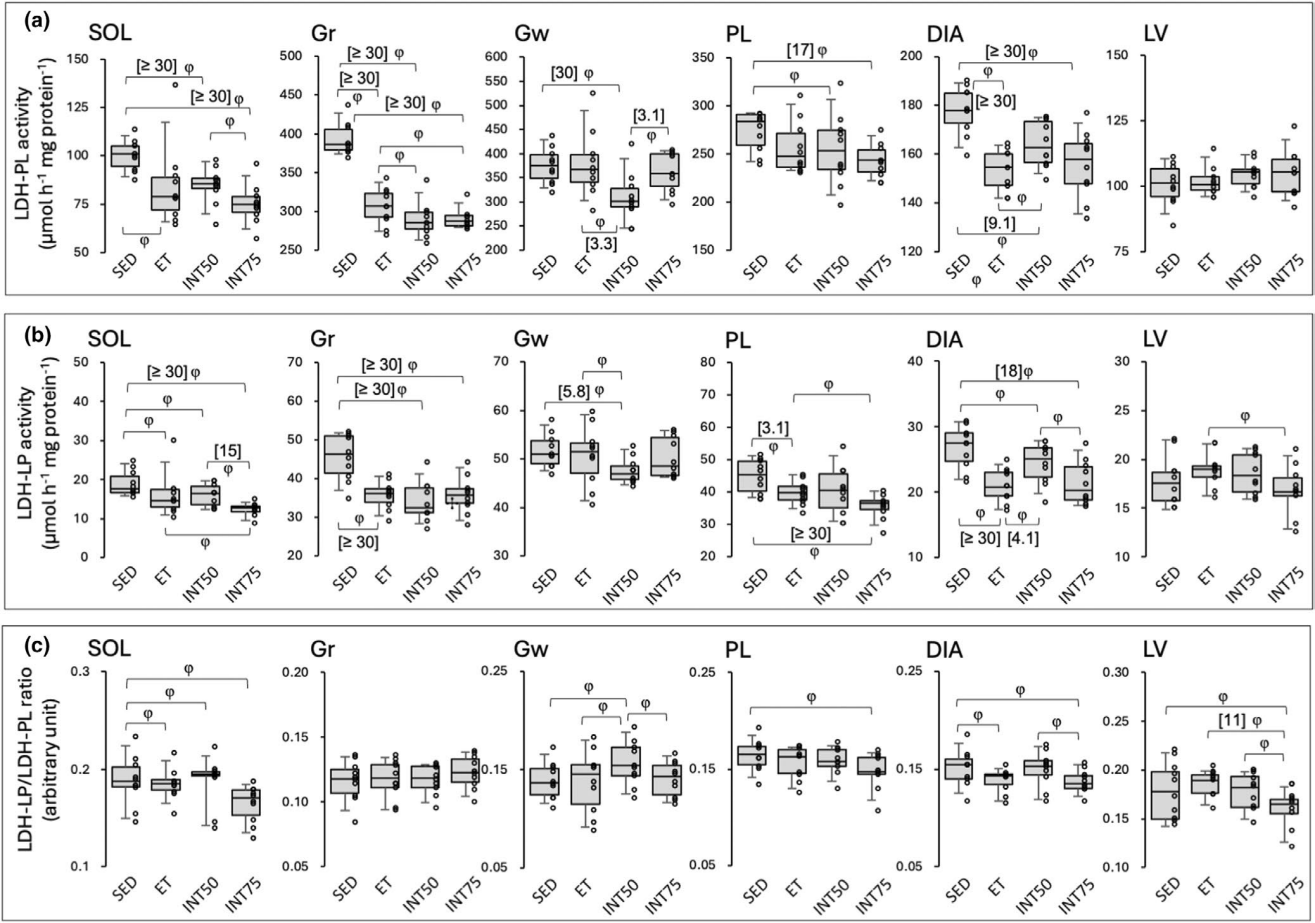
Enzyme activity values for LDH‐PL (a), LDH‐LP (b), and LDH‐LP/LDH‐PL ratio (c). Values are expressed as box and whisker plots with 5th, 25th, 50th, 75th, and 95th percentile. Dots are individual data points. Bayes factors are in parentheses. φ, The 95% confidence interval did not contain the mean value of target group for comparison.

**FIGURE 7:**
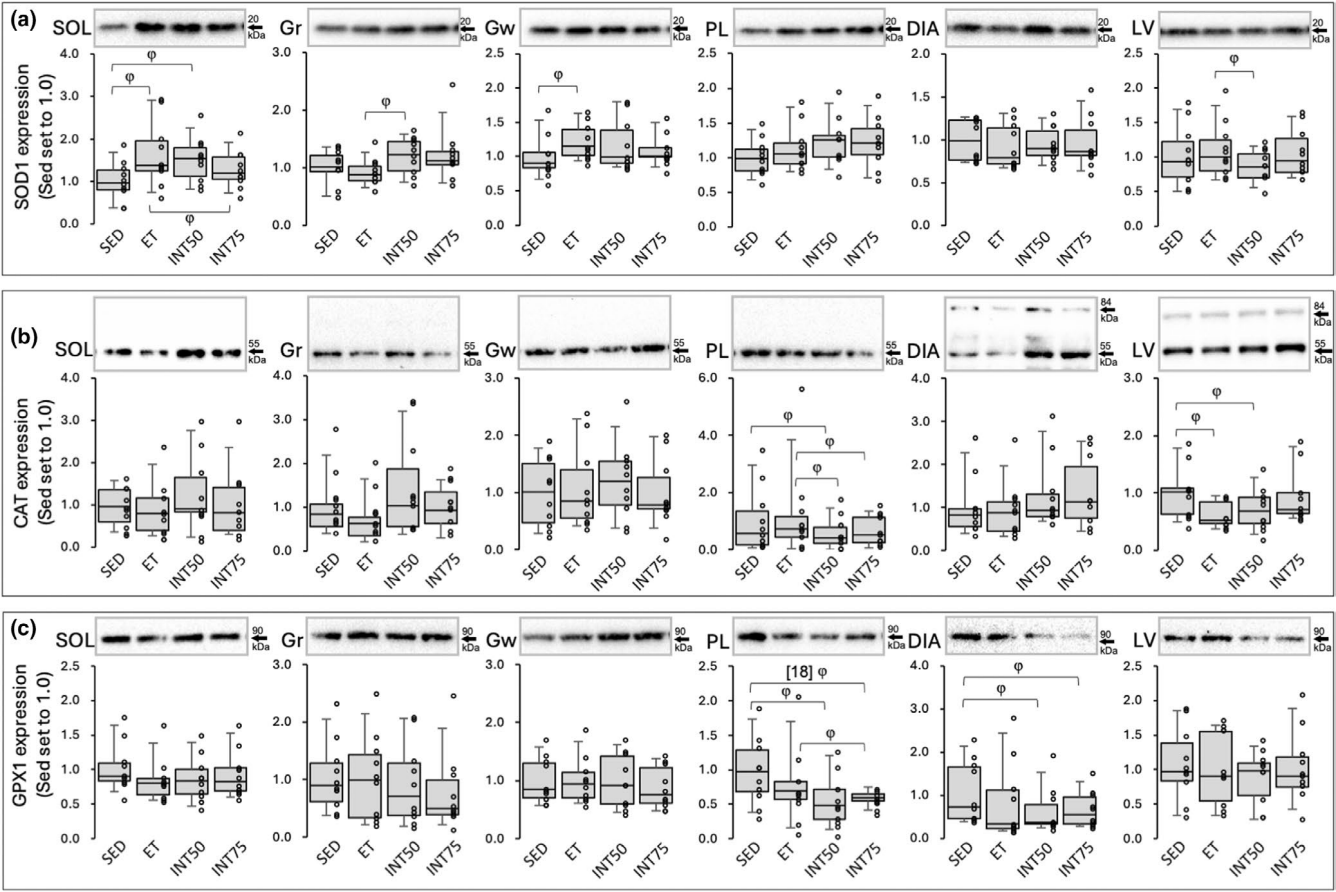
Protein levels for SOD1 (a), CAT (b), and GPX1 (c) at 48 h after the last exercise session. The densities of the specific bands were normalized to the densities of all protein bands in each lane on the membrane (Gilda & Gomes, 2013). Subsequently, the normalized densities of the bands were further normalized to the same sample that was run on every gel and transferred to every membrane, as previously reported (Suzuki, 2021). Representative blot images for SOD1, CAT, and GPX1 are shown in Figures S5–S7, S8–S10, and S11–S13, respectively. Values are expressed as box and whisker plots with 5th, 25th, 50th, 75th, and 95th percentile. Dots are individual data points. Bayes factors are in parentheses. φ, The 95% confidence interval did not contain the mean value of the target group for comparison.

**FIGURE 8:**
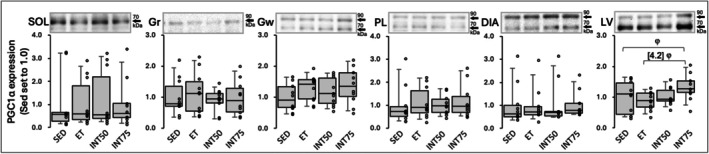
Protein levels for PGC1α at 48 h after the last exercise session. The densities of the specific bands were normalized to the densities of all protein bands. Representative blot images are shown in Figures S14–S16. Values are expressed as box and whisker plots with 5th, 25th, 50th, 75th, and 95th percentile. Dots are individual data points. Bayes factors are in parentheses. φ, the 95% confidence interval did not contain the mean value of the target group for comparison.

The present study employed Bayesian data analysis for statistical significance testing. All statistical analyses were performed using JASP (version 0.96). To identify specific differences between the four experimental groups, Bayesian post hoc pairwise comparisons were conducted. To protect against the inflation of false positives across the six pairwise tests, posterior probabilities were automatically adjusted for multiple comparisons utilizing the default Westfall‐based prior odds correction implemented in JASP (van Doorn et al., 2021). If the Bayes factor (BF, specifically BF10 in JASP) was greater than 3.0, the study confirmed the difference as statistically significant. Additionally, when the 95% confidence interval (CI) values did not include the mean value of the target group for comparison, the differences were considered biologically important (Du Prel et al., 2009; Gardner & Altman, 1986) and were described in the text as substantial or considerable changes. Data are presented as individual plots along with box and whisker plots that include the 5th, 25th, 50th, 75th, and 95th percentiles in Figures 2, 3, 4, 5, 6, 7, 8. In tables, data are expressed as means ± standard deviation (SD).

#### 2.7.2 Correlations

Pearson's product–moment correlation coefficients (*r*) were used to establish correlations between total work values and enzyme activity values. A correlation was calculated using data from the ET, INT50, and INT75 groups, as this study focused on the differences between these three exercise groups.

## 3 RESULTS

### 3.1 Voluntary wheel training

During 7 weeks of voluntary training, daily running distance increased gradually from week‐1 to week‐4. But the distance significantly decreased on the 6‐week (BF ≥30, vs. the 3rd and 4th week). However, on the final 7th week, run distance was recovered to the levels comparable to the 3rd and 4th week, presumably via supplemented treadmill run applied on the 5th to 7th week shown in the Figure S1.

### 3.2 Body mass and maximal exercise capacity

After 7 weeks of voluntary wheel running, the body weights of the ET, INT50, and INT75 groups were significantly lower than those of the SED group (BF ≥3.0, Table 1). Following 4 weeks of treadmill training with or without intermittent hyperoxic exposure, the organ mass‐to‐body mass ratio showed substantial increases in the three training groups than in the SED group (Table 1).

**TABLE 1:** Body and organ mass values.

	SED (*n* = 10)	ET (*n* = 10)	INT50 (*n* = 10)	INT75 (*n* = 10)
Body mass (g)				
After voluntary wheel run	40.0 ± 1.1 [38.2–42.0]	35.5 ± 2.0 §ε [31.4–38.2]	37.7 ± 2.5 §¶ψ [32.8–41.5]	36.4 ± 2.4 §ε [32.4–40.0]
After treadmill training	42.8 ± 1.3 ω [40.4–44.6]	37.0 ± 2.5 §εα [32.3–40.1]	38.6 ± 2.7 §εβ [34.7–43.0]	37.8 ± 2.5 §εβ [34.0–42.6]
Organ mass (mg)				
Soleus	9.50 ± 1.1 [7.7–11.0]	10.6 ± 1.0 § [9.0–12.4]	10.8 ± 1.3 § [8.9–12.9]	10.6 ± 1.3 § [7.6–12.2]
Plantaris	21.1 ± 1.7 [19.0–24.7]	21.7 ± 2.6 [18.8–27.3]	20.9 ± 2.6 [18.1–27.1]	22.2 ± 2.6 [17.9–26.5]
Gastrocnemius	180.2 ± 10.0 [170.1–203.0]	177.1 ± 11.6 § [164.9–200.8]	175.3 ± 16.8 [156.0–214.2]	170.0 ± 11.2 § [146.4–185.1]
Whole heart	165.6 ± 8.4 [156.6–183.9]	179 ± 15.8 § [149.6–205.5]	177 ± 19.6 [147.2–212.2]	177.1 ± 18.7 [149.3–205.8]
Left ventricle	118.0 ± 7.4 [109.4–132.1]	128.4 ± 11.4 § [110.9–147.5]	129.1 ± 15.5 [106.3–152.7]	129.5 ± 11.8 §ψ [115.1–149.8]
Organ mass‐to‐body mass ratio (mg g^−1^)				
Soleus	0.22 ± 0.02 [0.18–0.25]	0.29 ± 0.02 §ε [0.26–0.31]	0.28 ± 0.03 §ε [0.24–0.31]	0.28 ± 0.03 §ε [0.21–0.30]
Plantaris	0.49 ± 0.04 [0.43–0.56]	0.59 ± 0.10 §ψ [0.48–0.85]	0.54 ± 0.04 §¶ψ [0.48–0.63]	0.59 ± 0.1 §ε [0.53–0.74]
Gastrocnemius	4.2 ± 0.32 [3.81–4.87]	4.81 ± 0.5 §ψ [4.25–6.22]	4.54 ± 0.3 §¶ [4.04–4.98]	4.51 ± 0.3 §¶ [4.02–5.13]
Whole heart	3.87 ± 0.15 [3.72–4.15]	4.84 ± 0.4 §ε [4.30–5.34]	4.58 ± 0.4 §¶ε [3.93–5.28]	4.68 ± 0.2 §¶ε [4.39–5.06]
Left ventricle	2.76 ± 0.15 [2.51–2.98]	3.47 ± 0.3 §ε [3.03–3.89]	3.02 ± 1.1 §ε [2.83–3.80]	3.42 ± 0.2 §ƒε [3.20–3.64]

*Note*: Values are presented as means ± SD with range in brackets. ψ, δ, and ε, the difference of mean values was significantly different from the SED group at BF ≥3.0, ≥10, and ≥30. α, β, and ω, the difference of mean values was significantly different from the pre‐treadmill training at BF ≥3.0, ≥10, and ≥30. §, ¶, and ƒ, the 95% confidential interval did not contain the mean value of the SED, ET, and 50INT groups, respectively.

After 7 weeks of voluntary wheel running, total work values, obtained at “Pre‐testing” showing in Figure 2a, in the training group were significantly greater, by 7.7‐fold (BF ≥30), than those in the SED group. After 4 weeks of hybrid training, total work values in the ET group did not significantly increase (0.97‐fold, BF = 0.33). In contrast, the INT50 (1.15‐fold, BF = 3.0) and INT75 (1.17‐fold, BF ≥30) groups experienced a significant increase in total work values after hybrid training (Figure 2b). Additionally, total work values in the INT75 group, but not in the INT50, were significantly greater than those in the ET group (1.22‐fold, BF = 9.1).

### 3.3 Enzyme activity

In SOL, COX levels were unchanged after exercise training with and without intermittent hyperoxia. In Gr, COX levels were significantly higher in the three training groups compared to the SED group (BF ≥5.0, Figure 3a) and showed a significantly higher value in the INT50 group than in the ET group (by 15%, BF = 17.9). In Gw, COX levels were significantly greater only in the INT75 group than in the SED group (BF = 25). Moreover, COX values in Gw were significantly correlated with total work values (*r* = 0.34, Table 4). In LV, COX values in the INT75 group were significantly and substantially lower than those in the other three groups (BF ≥3.0).

In DIA, CS levels were significantly higher in the INT75 group than in the ET group (BF = 5.8, Figure 3b). CS values in DIA showed a significant positive correlation with total work values (*r* = 0.53, *p* < 0.05, Table 4).

CPT2 activity levels were significantly greater in the INT75 group than in the other three groups in DIA (BF ≥5.0, Figure 4a) and in LV (BF ≥4.6). HAD levels in DIA were significantly and substantially higher, respectively, in the INT75 group than in the ET (BF ≥30) and INT50 (CI: 1.01–1.27) groups (Figure 4b). HAD values in DIA were significantly correlated with total work values (*r* = 0.37, *p* < 0.05, Table 4). In Gw, HAD levels were significantly and substantially higher, respectively, in the INT75 group than in the ET (BF = 3.8) and INT50 (CI: 1.06–1.42) groups (Figure 4b). In LV, HAD values in the INT50 group were significantly and substantially lower, respectively, than in the SED (BF = 22) and ET (CI: 0.88–0.97) groups.

PDHc activity levels in PL were significantly greater in the INT75 group than in the ET (2.9‐fold, BF ≥30) and INT50 (2.2‐fold, BF ≥30) groups (Figure 5a). In Gr, PDHc activity levels were significantly greater in the INT50 (by 22%, BF ≥30) and INT75 (by 15%, BF = 8.1) groups than in the ET group. In LV, PDHc levels were markedly greater in the INT50 (by 22%, BF = 12.5) and INT75 (by 14%, CI: 1.03–1.26) groups than in the ET group. PDHc activity values showed positive correlations with maximal work values in PL (*r* = 0.43, *p* < 0.05), Gr (*r* = 0.38, *p* < 0.05), and LV (*r* = 0.32, *p* < 0.05, Table 4).

PFK activity levels in DIA were significantly higher in the INT75 group than in the ET (by 31%, BF = 3.4) and INT50 groups (by 16%, BF ≥30, Figure 5b). In Gr, PFK levels in the INT75 group were markedly higher than those in the INT50 (by 47%, BF = 20.9) and ET (by 27%, BF = 2.6, CI: 1.08–1.44) groups. In SOL, PFK activity levels were significantly lower in the INT75 group than in the other three groups (BF ≥8.0).

In the ET group, LDH‐PL levels were significantly lower than in the SED group in Gr (by 22%, BF ≥30) and DIA (by 14%, BF ≥30, Figure 6a). LDH‐PL activity levels in Gw were significantly lower in the INT50 group than in the other three groups (BF ≥3.0). In Gr, LDH‐PL levels were significantly lower in the three training groups than in the SED group.

In the ET group, LDH‐LP values were significantly lower than the SED group in Gr (by 21%, BF ≥30), PL (by 11%, BF ≥3.0), and DIA (by 22%, BF ≥30, Figure 6b). In DIA, LDH‐LP activity levels were significantly higher in the INT50 group than in the ET group (by 17%, BF = 4.1). LDH‐LP levels in SOL were markedly lower in the INT75 group than in the ET (by 35%, CI: 0.59–0.72) and INT50 (by 23%, BF = 14.6) groups.

In LV, the LDH‐LP/PL ratio levels were markedly lower in the INT75 group than in the ET and INT50 groups (by 14%, BF = 10.7 and by 9.7%, CI: 0.82–0.98, respectively, Figure 6c). The LDH‐LP/PL ratio values in LV were significantly lower in the INT75 group than in the ET group (by 22%, BF = 3.4). Thus, in the INT75 group, lactic acid utilization was likely diminished in highly oxidative muscle and the heart.

### 3.4 Protein levels

Protein abundance levels were determined by analyzing tissue samples collected 48 h after the final exercise capacity test.

Protein levels of SOD1 in SOL were substantially higher in the ET (1.6‐fold, CI: 1.08–2.18) and INT50 (1.5‐fold, CI: 1.12–1.88) groups than in the SED group (Figure 7a). In contrast, SOD1 levels were considerably lower in the INT75 group than in the ET group in SOL (0.78‐fold, CI: 0.59–0.97). SOD1 expression values in the INT50 group were substantially higher in Gr (1.3‐fold, CI: 1.05–1.56), whereas the values were considerably lower in LV (0.86‐fold, CI: 0.64–0.95) when compared to those in the ET group.

In PL, CAT expression levels were considerably lower in both the INT50 (0.49‐fold, CI: 0.17–0.81) and INT75 (0.55‐fold, CI: 0.24–0.87) groups than in the ET group (Figure 7b). CAT levels in LV were notably lower in the ET (0.62‐fold, CI: 0.45–0.78) and INT50 (0.73‐fold, CI: 0.46–0.99) groups than in the SED group.

In PL, GPX1 expression levels were significantly lower in the INT75 group compared to the ET (0.76‐fold, CI: 0.66–0.87) and Sed (0.59‐fold, BF = 3.3) groups (Figure 7c). GPX levels were also lower in the INT50 group (0.56‐fold, CI: 0.28–0.84) than in the SED group.

PGC1α expression levels in LV were markedly higher in the INT75 group than in the SED (1.4‐fold, CI: 1.03–1.64) and ET groups (1.5‐fold, BF = 4.2, Figure 8).

### 3.5 Muscle fiber‐type composition

In the INT75 group, the proportion of type I and type II fibers in GrM was significantly higher and lower, respectively, than in both the SED and INT50 groups (BF ≥3.0, Table 2). Additionally, the proportion of type IIA fibers in GrM was significantly greater in the INT75 group than in the SED group (BF ≥10). In the INT50 group, the proportions of type IIA and type II fibers in GrM were significantly higher than in the SED and INT75 groups, respectively (BF ≥3.0). Thus, exercise under intermittent hyperoxia at 50% O_2_ did not result in an exercise‐induced increase in the proportion of type I fibers. In contrast, the 75% O_2_ condition significantly reduced highly glycolytic type II fibers in oxidative muscle.

**TABLE 2:** Fiber type composition values (%).

		SED (*n* = 10)	ET (*n* = 10)	INT50 (*n* = 10)	INT75 (*n* = 10)
SOL	I	50.5 ± 4.6	58.6 ± 7.1 §	56.7 ± 10.4	56.7 ± 6.7 ψ§
	IIA	43.7 ± 5.0	40.4 ± 7.6	42.7 ± 10.3	43.0 ± 6.7
	II	5.8 ± 4.5	1.06 ± 1.7 ψ	0.58 ± 1.3 δ	0.27 ± 0.6 δ
PL	I	6.9 ± 4.8	5.5 ± 3.4	5.8 ± 3.1	5.1 ± 3.4
	IIA	45.6 ± 6.6	55.3 ± 8.6 ψ§	51.9 ± 5.2 §	53.9 ± 3.4 δ§
	II	47.5 ± 9.0	39.2 ± 10.6 §	42.2 ± 6.3 §	41.0 ± 5.5 §
GrL	I	17.5 ± 3.0	16.3 ± 2.9	15.0 ± 5.6	16.4 ± 4.2
	IIA	42.7 ± 6.9	49.4 ± 2.7 §	53.0 ± 12.9 §	50.5 ± 5.5 ψ§
	II	39.8 ± 7.9	34.3 ± 3.8 §	32.0 ± 17.4	33.1 ± 6.2 §
GrM	I	27.6 ± 10.2	34.9 ± 8.6 §	26.9 ± 8.6 ¶	38.9 ± 6.2 ψπ§ƒ
	IIA	42.4 ± 6.5	47.5 ± 6.9 §	52.0 ± 9.3 ψ§	52.3 ± 6.5 δ§¶
	II	30.0 ± 11.4	17.6 ± 10.9	21.2 ± 12.6	8.8 ± 8.8 εω
GW	ΙΙ	100	100	100	100

*Note*: Values are presented as means ± SD. ψ, δ, and ε, the difference of mean values was significantly different from the SED group at BF ≥3.0, ≥10, and ≥30. κ, λ, and θ, the difference of mean values was significantly different from the ET group at BF ≥3.0, ≥10, and ≥30. ω, and π, the difference of mean values was significantly different from the 50INT group at BF ≥3.0, and ≥10. §, ¶, and ƒ, the 95% confidential interval did not contain the mean value of the SED, ET, and 50INT groups, respectively.

### 3.6 Capillarization

Values of the capillary‐to‐fiber ratio (C/F) in PL were significantly and considerably higher in the INT75 group compared to the SED (12.6‐fold, BF ≥30) and ET (1.1‐fold, CI: 1.01–1.16) groups, respectively (Table 3). Capillary density (CD) values in PL were significantly higher in the INT50 (1.2‐fold, BF ≥3.0) and INT75 (1.4‐fold, BF ≥30) groups, but not in the ET (1.1‐fold) group, compared to the SED group. The CD values in SOL were significantly higher in the INT75 group compared to the SED (1.3‐fold, BF ≥10) and INT50 (1.1‐fold, CI: 1.04–1.24) groups. Thus, exercise under short‐duration intermittent hyperoxia promotes exercise‐induced capillary growth in the hindlimb of highly trained mice.

**TABLE 3:** Capillary‐to‐fiber ratio and capillary density values.

	SED (*n* = 10)	ET (*n* = 10)	INT50 (*n* = 10)	INT75 (*n* = 10)
Capillary‐to‐fiber ratio				
SOL	1.67 ± 0.19	1.90 ± 0.21 ψ§	2.01 ± 0.15 ε§	2.12 ± 0.18 ε§
GrL	1.84 ± 0.19	2.08 ± 0.29 §	2.13 ± 0.21 δ§	2.12 ± 0.10 δ§
GrM	1.78 ± 0.14	1.88 ± 0.18	1.99 ± 0.19 §	2.04 ± 0.17 §
Gw	0.98 ± 0.09	0.97 ± 0.11	1.00 ± 0.11	1.00 ± 0.07
PL	1.59 ± 0.17	1.89 ± 0.20 δ§	2.01 ± 0.20 ε§	2.05 ± 0.18 ε§¶
Capillary density (number per mm^2^)				
SOL	979.1 ± 154.1	1150.0 ± 226.3 §	1103.6 ± 142.5 §	1255.1 ± 156.4 δ§≠
GrL	1048.3 ± 168.6	1105.5 ± 172.5	1177.5 ± 206.0	1231.5 ± 324.7
GrM	921.2 ± 127.7	1192.9 ± 411.8	1122.2 ± 237.0 §	1123.2 ± 132.2 κ§¶
Gw	422.9 ± 66.8	399.8 ± 58.3	396.4 ± 87.5	397.1 ± 50.6
PL	885.7 ± 199.5	985.2 ± 337.6	1106.9 ± 135.6 ψ§¶	1224.5 ± 238.0 ε§¶

*Note*: Values are presented as means ± SD. ψ, δ, and ε, Τhe difference of mean values was significantly different from the SED group at BF ≥3.0, ≥10, and ≥30. κ, λ, and θ, Τhe difference of mean values was significantly different from the ET group at BF ≥3.0, ≥10, and ≥30. §, ¶, and ≠ The 95% confidential interval did not contain the mean value of the SED, ET, and 50INT groups, respectively.

## 4 DISCUSSION

The body weight showed a significant increase after 4 weeks of exercise training, both with and without intermittent hyperoxia (BF ≥3.0, Table 1). Thus, it is likely that the present hybrid exercise training under intermittent hyperoxia did not cause physical and/or mental distress in well‐trained mice.

In the present study, 7 weeks of voluntary wheel training significantly improved endurance exercise capacity (Figure 2a). Exercise training with a voluntary wheel device enhanced endurance capacity after 4 (Kim et al., 2023; Wada et al., 2015) and 8 weeks (Bell et al., 2019) in mice. As previously reported (Suzuki, 2025), voluntary running distance increased gradually during the first 3 weeks but tended to decline in the 5th week and beyond. To prevent this decline, additional treadmill running was implemented from the 5th to the 7th week, as shown in Figure S1.

The present study demonstrated that hybrid exercise training, combining endurance and interval exercise with short‐duration intermittent hyperoxic exposure (referred to as INT training), had an additive effect on improving endurance capacity at a higher oxygen concentration of 75% O_2_, but not at 50% O_2_, in well‐trained mice (Figure 2b). However, the exercise capacity increase in the INT75 group (by 22%) was lower than that of a previous study (by 27%) using 30% O_2_ (Suzuki, 2025). Thus, the additive effects of intermittent hyperoxia may not enhance endurance capacity depending on the level of oxygen concentration applied.

In Thoroughbred horses, after a single bout of high‐intensity exercise under hyperoxia (26% O_2_) induced responses of genes related to lysosomal activity, whereas hypoxic exercise (16% O_2_) triggered hypoxia‐responsive gene expression (Takahashi et al., 2025). A previous work (Suzuki, 2024) showed intermittent, but not continuous, hyperoxia (30% O_2_) at rest enhanced hypoxia‐responsive gene levels (VEGFA and PFK) as well as gene levels of peroxisome proliferator‐activated receptors. Thus, the INT training improved metabolic enzyme levels and capillary profiles (Suzuki, 2025).

INT75 training caused significant changes in metabolic properties in DIA. It promoted fatty acid metabolism, indicated by increased activity levels of HAD and CPT2 in DIA (Figure 4). Additionally, INT75 elevated the levels of CS, the rate‐limiting enzyme of the citric cycle, and PFK, an enzyme in the glycolytic pathway in DIA. The activity levels of these four enzymes (HAD, CPT2, CS, and PFK) were significantly correlated with maximal work values (Table 4). Thus, in respiratory muscle, INT75 training may enhance both fatty acid and carbohydrate utilization during exercise. In a previous study using 30% O_2_, INT training increased CS levels but downregulated PFK levels in DIA (Suzuki, 2025).

**TABLE 4:** Correlation with total work values.

	Explanatory variable	BF	*r*	*p*
Gr	PHDc	3.5	0.381	<0.05
GW	CS	2.2	0.342	<0.05
	COX	3.1	0.372	<0.05
PL	PHDc	6.2	0.426	<0.05
	LDH‐PL	9.0	−0.452	<0.05
DIA	PFK	2.7	0.359	<0.05
	CS	31.5	0.526	<0.05
	CPT2	22.6	0.508	<0.05
	HAD	2.9	0.365	<0.05
LV	PHDc	1.8	0.323	<0.05
	CS	7.9	0.443	<0.05
	CPT2	5.0	0.41	<0.05

Abbreviations: BF, Bayes factor; p, NHST *p*‐value; *r*, Pearson's product monent correlation coefficient.

HAD levels were also markedly increased in Gw after INT75 (Figure 4b). Although blood flow to Gw was unchanged during exercise in untrained rats (Delp et al., 1989), blood flow to the fast‐twitch white region of the calf muscle increased by 40%–50% after exercise training (Mackie & Terjung, 1983). In mice, Gw muscle regions comprise approximately 40% of the calf cross‐sectional area at the mid‐belly (Suzuki, 2019). Thus, enhanced fatty acid utilization in respiratory muscle and in muscles with a large volume in the hind leg promoted endurance capacity. As previously reported (Suzuki, 2025), activity levels of HAD and CPT2 were not significantly elevated in DIA after INT with 30% O_2_.

For the INT50, CPT2, and PFK levels were upregulated in DIA (Figures 4a and 5b, respectively), while CS levels remained unchanged (Figure 3b). Thus, fatty acid flux in mitochondria and carbohydrate breakdown improved only in the respiratory muscle after the INT50.

PDHc is a complex of three enzymes that converts pyruvate into acetyl‐CoA, which is then used in the citric acid cycle, linking the glycolytic pathway to the citric acid cycle. During cycling exercise, PDHc activation in the human vastus lateralis muscle was directly proportional to relative aerobic power output (% of maximal oxygen consumption) (Spriet & Heigenhauser, 2002). In rats, voluntary wheel running for 8 weeks did not change PDHc activity levels in skeletal muscle (Nakai et al., 1999). Thus, the changes in PDHc activity levels observed in the present study likely reflect the effects of hybrid training with both INT50 or INT75. The increased PDHc activity levels after the INT75 training likely enable individuals to perform exercises at higher intensities. After the INT75, PDHc activity levels were upregulated in Gr and PL (Figure 5a). The increased PDHc activity levels likely enable individuals to perform exercises at higher intensities. In well‐trained mice, the hybrid training used in this study did not improve PDHc levels (Suzuki, 2019; Suzuki, 2025). Therefore, the INT75 training likely promotes glycogen utilization by upregulating PDHc activity levels in both oxidative and glycolytic muscles in well‐trained mice. After the INT50, notable increases in PDHc levels were also observed in Gr and LV as observed in the INT75 (Figure 5a). Thus, INT with 50% O_2_ or higher likely promotes PDHc levels in oxidative and glycolytic muscles and the heart.

COX (complex IV) is the final step of the electron transport chain, receiving electrons from cytochrome c and irreversibly reducing oxygen to water (Kadenbach & Hüttemann, 2015). COX is the rate‐limiting enzyme of mitochondrial respiration and plays a crucial role in aerobic energy metabolism by regulating mitochondrial respiration (Ramzan et al., 2021). After 8 weeks of voluntary running, COX activity levels remained unchanged in the gastrocnemius muscle (Bell et al., 2019). Thus, changes in COX levels in the present study likely reflected the effects of ET or INT training.

Following the INT75 training, COX activity levels were significantly reduced in LV (Figure 3a). Transient hyperoxic reoxygenation after ischemia in cardiac muscle has been shown to decrease both COX activity and protein levels due to increased nitric oxide (NO) levels from inducible nitric oxide synthase (Arab et al., 2010). Cytosolic SOD levels increased to protect NO‐induced cellular damage (Arab et al., 2010). In the present study, SOD1 protein levels in LV at 48 h after the last exercise showed no significant difference among the four groups (Figure 7a). During exercise, nitrite/nitrate (NOx) levels in the heart were significantly lower by 33% compared to resting rats (Iemitsu et al., 2000). Therefore, it is likely that NO production did not increase during the present exercise under intermittent hyperoxia. Although the precise mechanisms underlying reduced COX activity levels in the heat have not been elucidated, periodic large fluctuations in PO_2_ likely suppress mitochondrial oxidative phosphorylation.

After the present INT75, PGC1α protein levels were significantly elevated in cardiac muscle (Figure 8). This upregulation is likely a compensatory response to restore COX levels. In the COX deficiency model, the Cohen (CDs) rat, COX activity levels were approximately half of those in control rats (Kogot‐Levin et al., 2016). In the present study, cardiac COX activity levels in the INT75 group were 0.49‐fold those in the SED group (Figure 3a). In fibroblasts derived from CDs rats, PGC1α protein levels were significantly higher (approximately 1.7‐fold) than in control rats (Kogot‐Levin et al., 2016). Overexpression of PGC1 led to a robust increase in mitochondrial biogenesis and mitochondrial respiratory chain activities, including COX (Viscomi et al., 2011). In the left ventricle, PGC1α protein levels were 1.5‐fold higher in the INT75 group than in the ET group (Figure 8). Thus, elevated PGC1α protein levels likely serve as a transcriptional coactivator to restore mitochondrial biogenesis. While more precise studies are needed, prolonged INT training at over 75% O_2_ may pose a risk of reduced mitochondrial biogenesis in cardiac muscle.

Eight isoforms of GPX exist, and all GPX enzymes require selenium as a cofactor, using reducing equivalents from GSH to reduce both H_2_O_2_ and organic hydroperoxides to form water or alcohol (Powers et al., 1994). The presence of multiple isoforms of GPX makes sense due to their different locations (Brigelius‐Flohé & Flohé, 2020). GPX1 is found in both mitochondria and the cytosol of muscle fibers (Brigelius‐Flohé & Flohé, 2020). Its main function appears to be the reduction of H_2_O_2_ and other soluble hydroperoxides at the expense of GSH (Powers et al., 1994). The amount of GPX in skeletal muscle fibers varies across fiber types. Highly oxidative fibers have the highest GPX activity, while muscle fibers with low oxidative capacity (type IIB and IIX) exhibit the lowest GPX levels in rodents (Powers et al., 1994).

In the present study, 48 h after the last exercise bout, GPX1 protein levels in PL were significantly reduced after the INT75 (Figure 7c). PL muscle consists of approximately 40% type II fibers (Table 2), so basal GPX1 levels are likely lower in PL than in other oxidative muscles, respiratory muscles, and the heart. In PL, although CS enzyme activity significantly increased after training with and without INT, COX activity levels remained unchanged after INT75 training (Figure 3a). In mice, genetic inactivation of GPX1 led to growth retardation, presumably due in part to reduced mitochondrial energy production resulting from increased oxidative stress (Esposito et al., 2000). Thus, the enhanced oxidative stress caused by the marked reduction in GPX1 protein levels likely inhibited COX expression levels in PL muscle.

Although the proportion of type I fibers in the ET and INT75 groups markedly increased in the highly oxidative GrM portion after hybrid exercise training, the INT50 training did not affect these proportions (Table 2). After 10 weeks of wheel running, the expression of type I myosin heavy chain did not change in the lower leg muscles (Schmitt et al., 2020). Consequently, the changes in type I fiber proportions observed in this study likely reflect the effects of hybrid training with the INTs. Hyperoxic exposure (50% O_2_, 1 h per day for 4 weeks) significantly shifted fiber type proportions in EDL muscle, decreasing type I and increasing type II fibers in STZ‐induced diabetic rats (Sugimoto et al., 2025). However, these fiber type shifts were not observed in the group exposed to 40% O_2_. Therefore, the lower proportion of type I and higher proportion of type II fibers in the INT50 group likely contributed to the inadequate increase in endurance exercise capacity.

In well‐trained mice, as used in this study, 4 weeks of hybrid training increased capillary supply compared to the SED group only in the PL muscle (Suzuki, 2019; Suzuki, 2021). Furthermore, intermittent hypoxic exposure (Suzuki, 2019) or hyperbaric exposure (Suzuki, 2021) at rest did not lead to a further increase in capillary supply. In a previous study, capillary supply in both oxidative (SOL and GrL) and glycolytic (PL) muscle portions was enhanced by INT training with 30% O_2_ (Suzuki, 2025). In the present study, however, the capillary‐to‐fiber (C/F) ratio values in the INT75 group, but not in the INT50 group, showed a considerable increase only in the PL muscle (by 8.4% compared to the ET group, CI: 1.01–1.16, Table 3). Thus, INT training with higher O_2_ concentration (>50%) may induce only a modest improvement in capillary supply in hind leg muscles.

The LDH enzyme exists as a tetramer and has five isomeric forms composed of different ratios of the two subunits, M and H (Markert, 1963), which are encoded by the LDHA and LDHB genes, respectively (Li, 1989). Unlike the LDHB gene, the LDHA gene contains hypoxia recognition sites in its promoter sequence, making it responsive to HIF1α (Semenza et al., 1994). Consequently, LDHA transcription is upregulated by acute hypoxia (Firth et al., 1995), while LDHB generally shows no significant response to hypoxia (Osis et al., 2021). In the present study, the LDH‐LP/‐PL ratio (Figure 6c) was observed, potentially indicating the H/M isozyme ratio. The INT75 training significantly decreased the LDH‐LP/‐PL ratio and notably lowered LDH‐LP activity in SOL and LV (Figure 6c). Although the present study did not measure metabolite concentrations precisely, this notion suggests that INT75 may reduce the use of lactate as fuel in highly oxidative muscle and the heart, potentially suppressing endurance exercise capacity. After the INT50, the capacity for lactate production from pyruvate (LDH‐PL activity levels) in the highly glycolytic Gw muscle portion, which may reflect glycolytic capacity, was significantly reduced (Figure 6a), likely hindering high‐intensity exercise.

In the present study, effects of INT training were evaluated by enzyme activity levels in male mice. In adult mice, at 56 and 140 days of age, activity levels of CS, COX, and HAD in skeletal muscles were shown to be lower in females than those in males (Takahashi et al., 2022). Thus, the present results concerning enzyme activity levels are limited to male mice.

## 5 CONCLUSION

In this study, male mice underwent 7 weeks of voluntary running, resulting in a 7.7‐fold increase in endurance exercise capacity. The trained mice then participated in INT training, which combines exercise with short‐duration intermittent hyperoxic intervention (50% [INT50] or 75% [INT75] O_2_) for 4 weeks. INT75 provided additional benefits for improving endurance capacity, while INT50 did not. INT75 training enhanced fatty acid metabolism, indicated by increased HAD and CPT2 activity levels in DIA. As previously reported (Suzuki, 2025), using 30% O_2_ for INT training, activity levels of these enzymes were not significantly elevated in DIA. Furthermore, INT75 increased the levels of CS, a rate‐limiting enzyme in the citric acid cycle, and PFK, a key enzyme in the glycolytic pathway, in DIA. Thus, INT75 training likely promotes fatty acid and carbohydrate utilization in respiratory muscles during exercise, enhancing endurance capacity. INT50 training modestly enhanced oxidative metabolism in highly oxidative tissues while reducing lactate production in glycolytic muscle. Additionally, it altered the fiber type proportion in oxidative muscle, resulting in lower type I and higher type II fibers. These changes likely explain why INT50 did not improve endurance capacity. Although the precise mechanisms remain unclear, these findings highlight that the effects of INT training vary based on the oxygen level used in well‐trained mice.

## AUTHOR CONTRIBUTIONS

**Junichi Suzuki:** Conceptualization; data curation; formal analysis; funding acquisition; investigation; methodology; project administration; resources; software; supervision; validation; visualization.

## FUNDING INFORMATION

This work was supported by JSPS KAKENHI Grant Number: JP23K10579; JP26K14331.

## CONFLICT OF INTEREST STATEMENT

None declared.

## ETHICS STATEMENT

All procedures were approved by the Animal Care and Use Committee of Hokkaido University of Education (No. 1, approved on 2025/3/31).

## Supporting information


Figures S1–S16.

## Data Availability

Data file that supports the present results are available at https://docs.google.com/spreadsheets/d/1MhqFx0BP5Sh52s3iZFGnGLql9fpetcbx/edit?usp=sharing&ouid=103780764902759483879&rtpof=true&sd=true.
