# Supplementary material for: Effects of different oxygen concentrations during intermittent hyperoxic training on endurance capacity in well‐trained male mice
Source: Physiol Rep. 2026 Jul 18;14(14):e71020. doi: 10.14814/phy2.71020 (PMC13379783; doi:10.14814/phy2.71020)

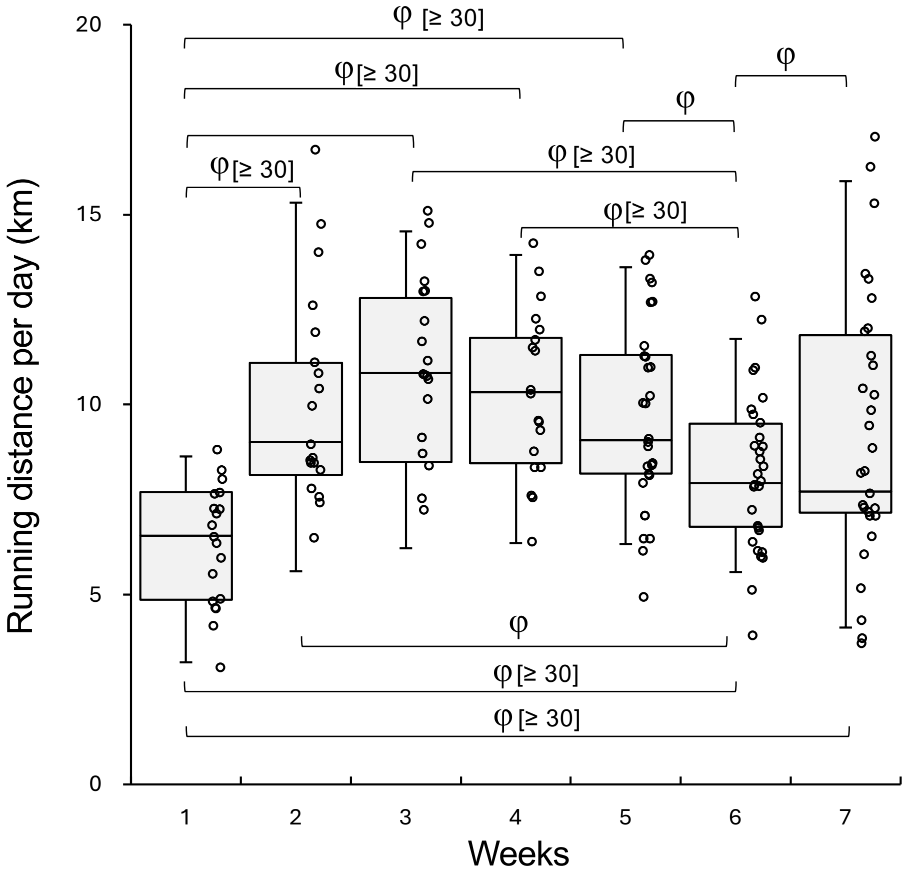

Figure S1 Running distance per day during voluntary wheel training
Values are expressed as box and whisker plots with 5th, 25th, 50th, 75th and 95th percentile. Dots in the figure indicate data for each mouse. Bayes factors are in parentheses. ϕ, The 95% confidential interval did not contain the mean value of target group for comparison.

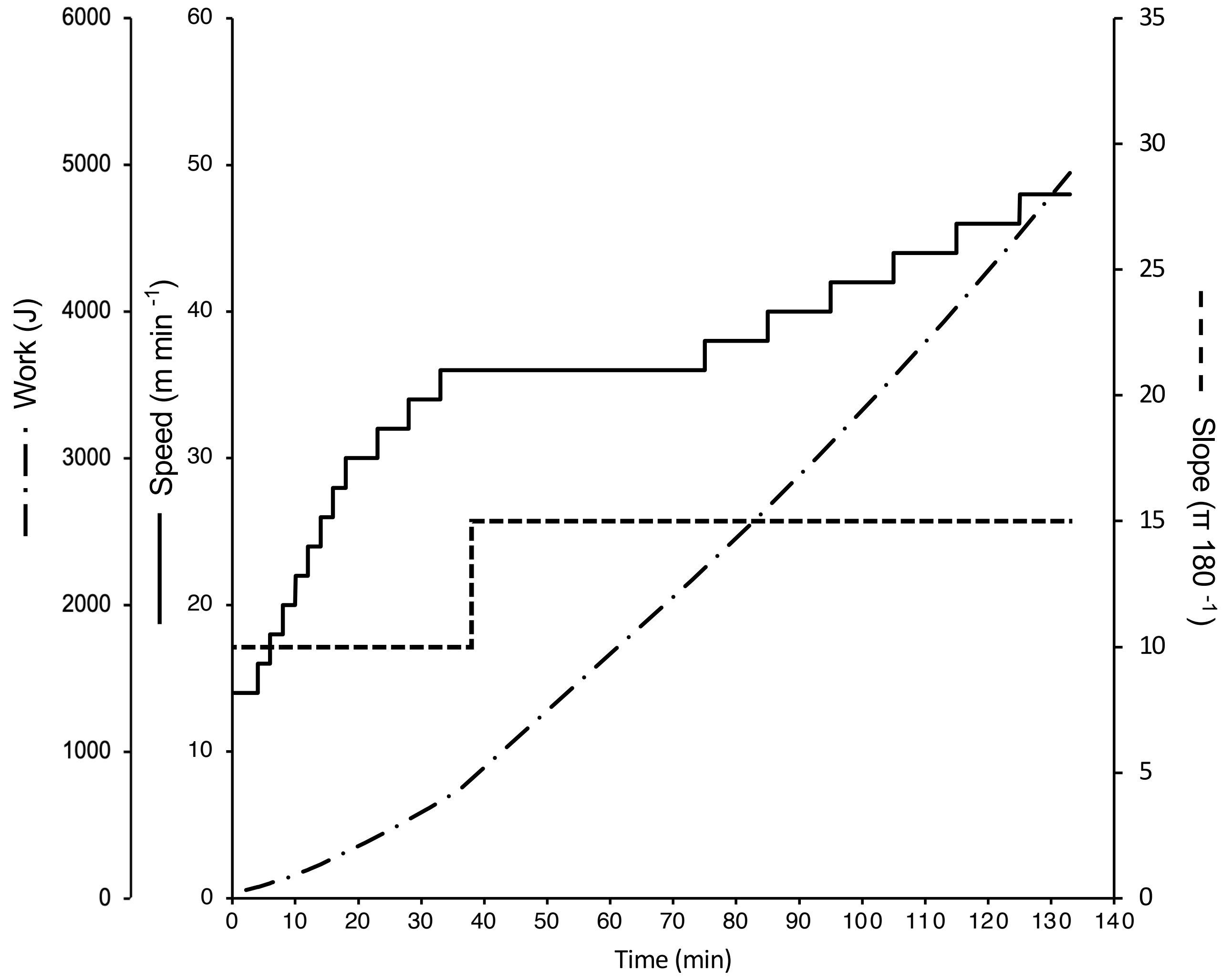

Figure S2 Graded ramp treadmill running protocol for the endurance capacity test. The values of work (J) in the figure are calculated for a body weight is 40 g.

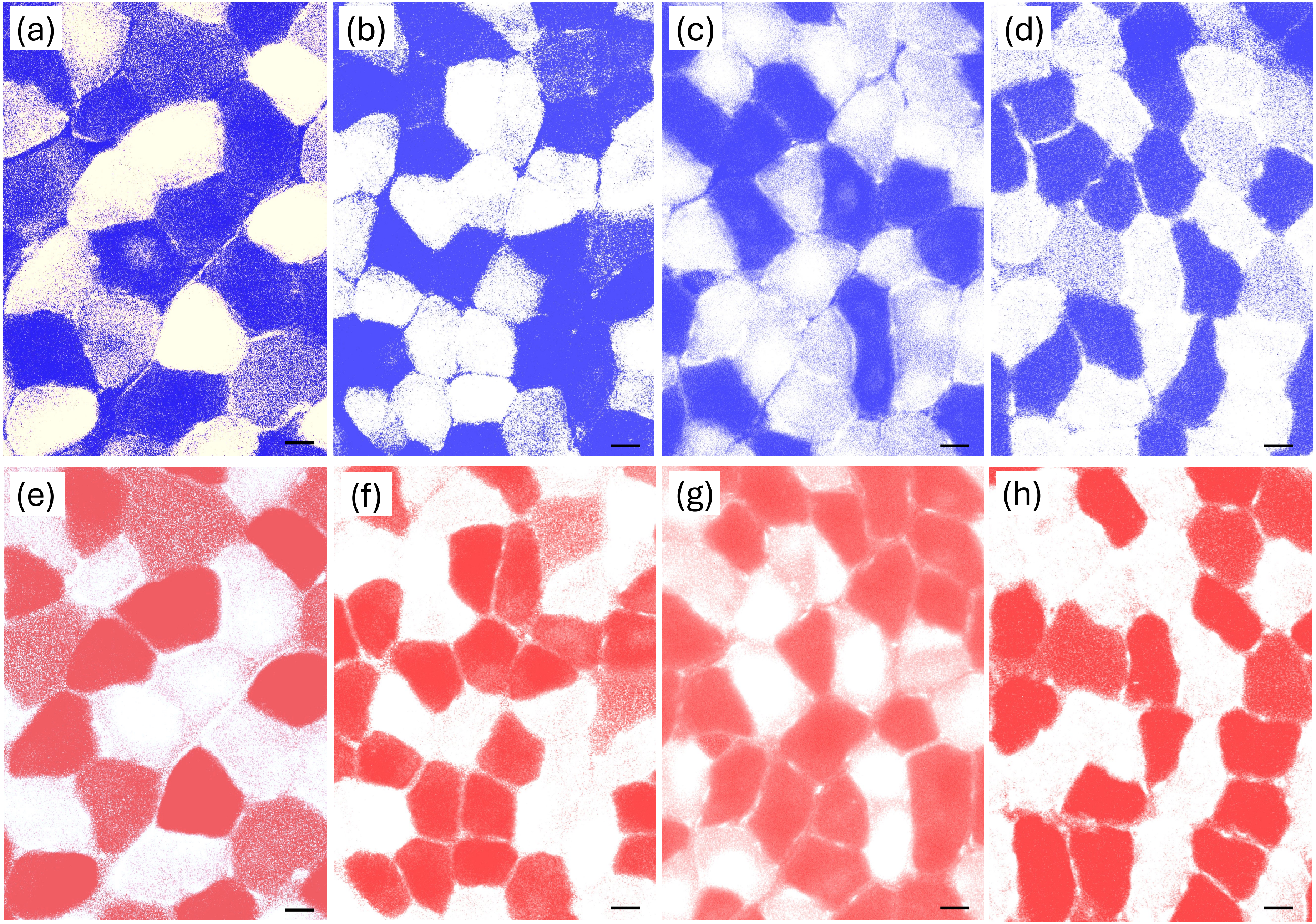

Figure S3 Representative immunofluorescent images of muscle fiber profiles for red portion of gastrocnemius. (a and e), SED; (b and f), ET; (b and f), INT50; (c and g), INT75 (d and h) groups. (a-d), type I fiber; (e-h), type IIA fiber. Horizontal bars represent 20 μm.

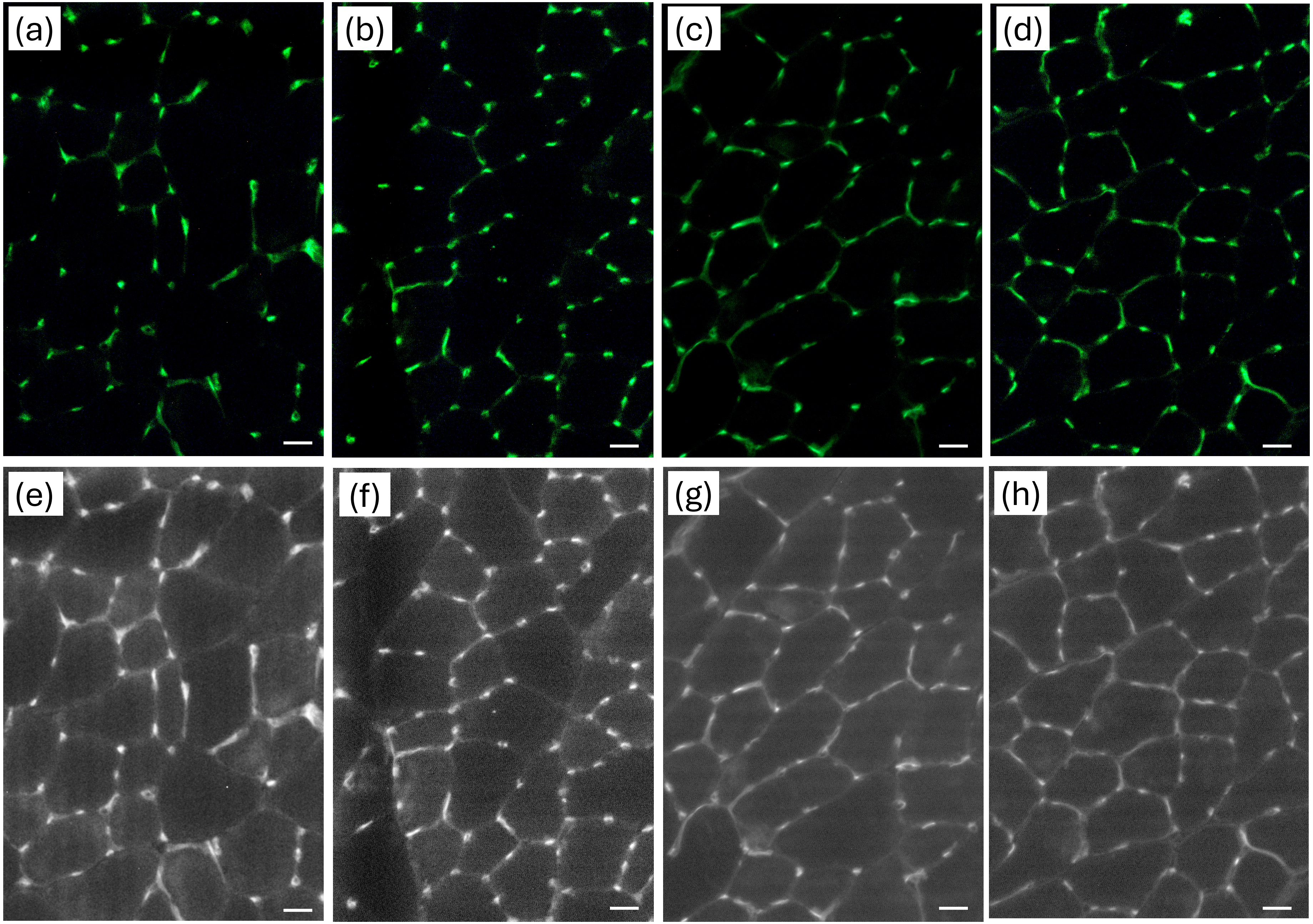

Figure S4 Representative images for capillary profiles (a-d) and muscle fiber profiles (e-h) of plantaris muscle (GrL) for SED (a and e), ET (b and f), INT50 (c and g), and INT75 (d and h) groups. Horizontal bars represent 20 µm.

Figure S5

Complete blot images shown in Figure

SOD1

The lanes used in the figure

Molecular weight marker: Precision Plus Protein™ All Blue Prestained Protein Standards #1610373

Total protein images after electrophoresis followed by trans-blot to membrane used as loading control

Specific bands of target protein detected after immunostaining

SOL

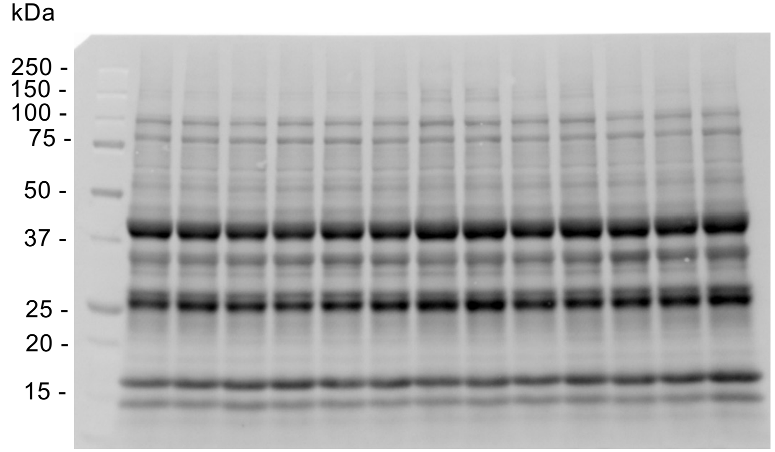

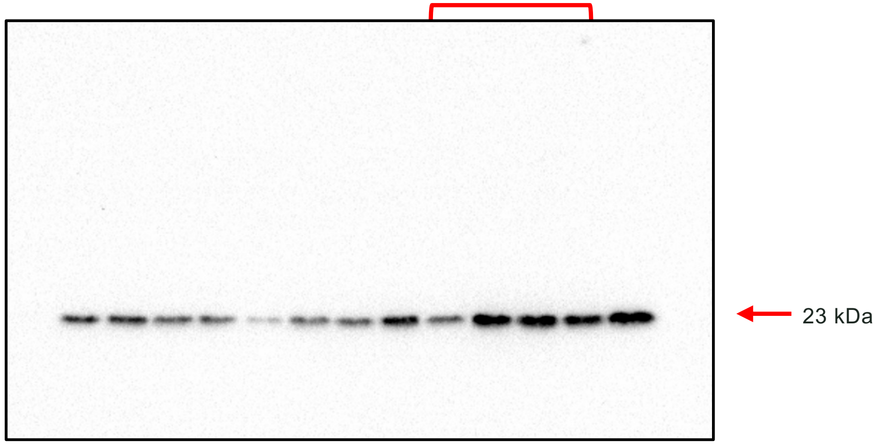

Gr

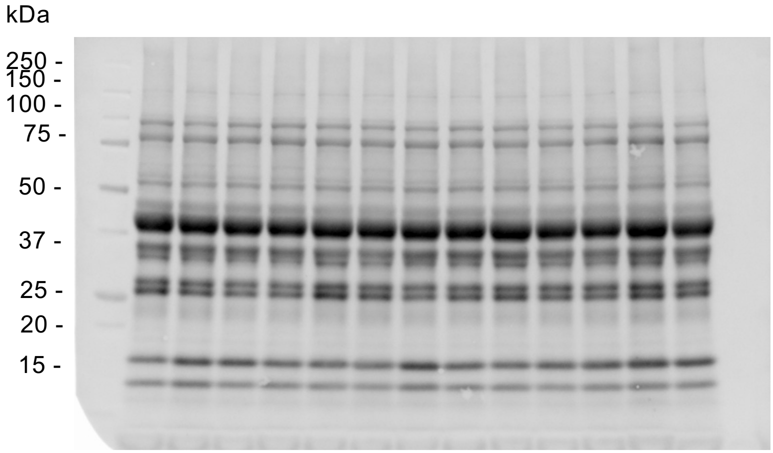

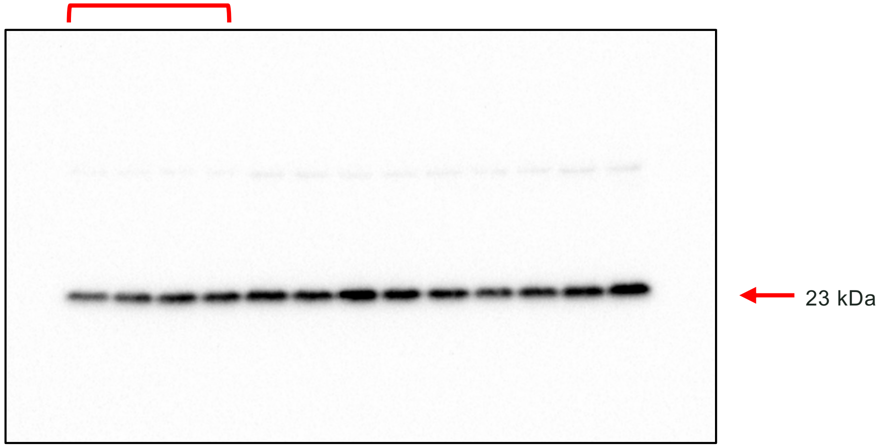

Figure S6

Molecular weight marker: Precision Plus Protein™ All Blue Prestained Protein Standards #1610373

Complete blot images shown in Figure

SOD1

The lanes used in the figure

Total protein images after electrophoresis followed by trans-blot to membrane used as loading control

Specific bands of target protein detected after immunostaining

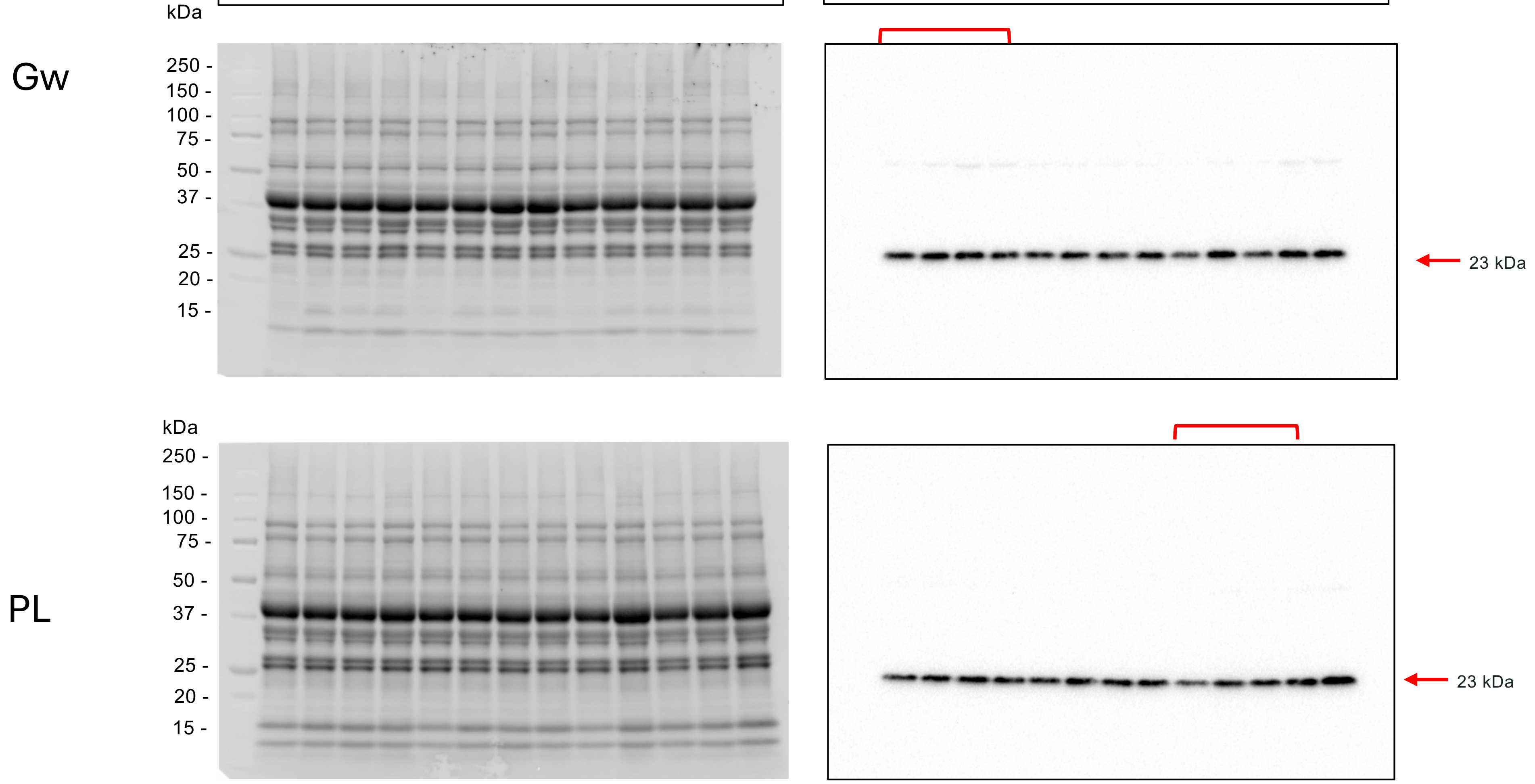

Figure S7

Molecular weight marker: Precision Plus Protein™ All Blue Prestained Protein Standards #1610373

Complete blot images shown in Figure

The lanes used in the figure

SOD1

Total protein images after electrophoresis followed by trans-blot to membrane used as loading control

Specific bands of target protein detected after immunostaining

DIA

kDa

250 -
150 -
100 -
75 -
50 -
37 -
25 -
20 -
15 -

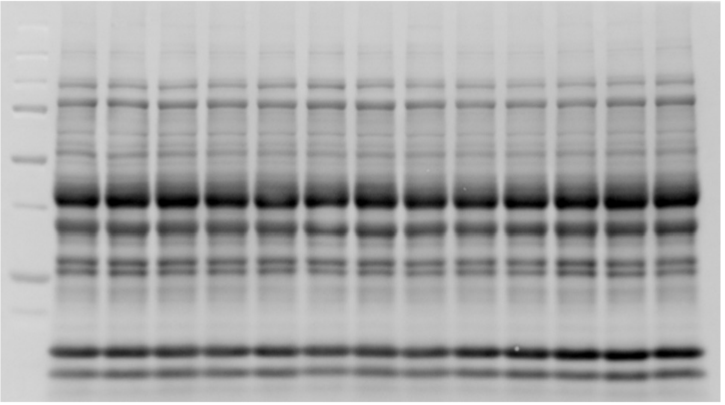

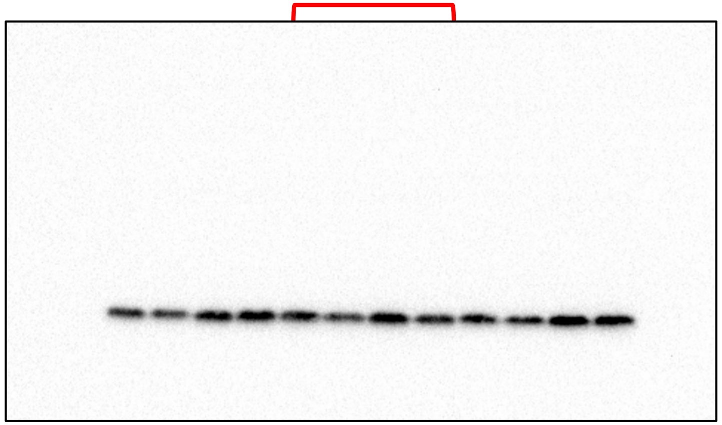

23 kDa

LV

kDa

250 -
150 -
100 -
75 -
50 -
37 -
25 -
20 -
15 -

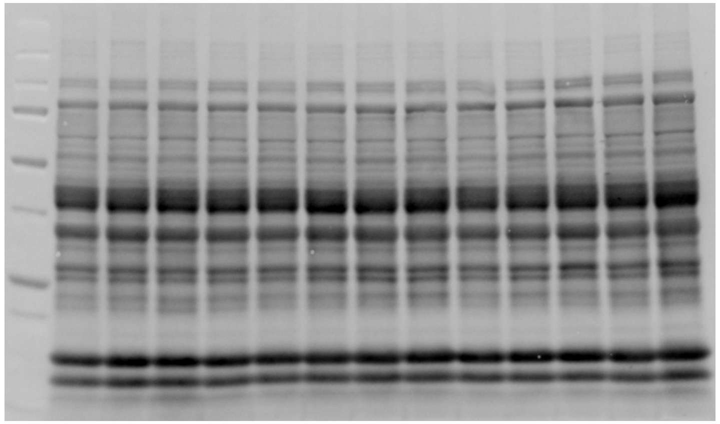

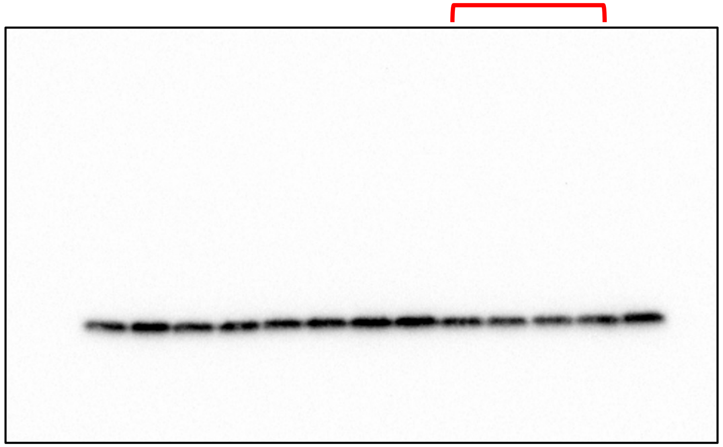

23 kDa

Figure S8

Molecular weight marker: Precision Plus Protein™ All Blue Prestained Protein Standards #1610373

Complete blot images shown in Figure

The lanes used in the figure

CAT

Total protein images after electrophoresis followed by trans-blot to membrane used as loading control

Specific bands of target protein detected after immunostaining

SOL

kDa

250 -
150 -
100 -
75 -
50 -
37 -
25 -
20 -
15 -

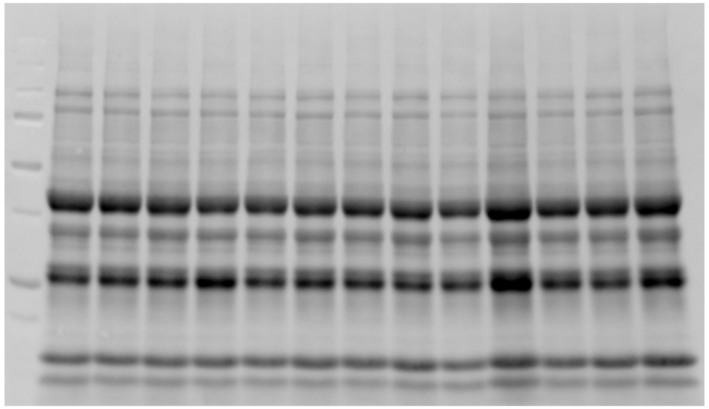

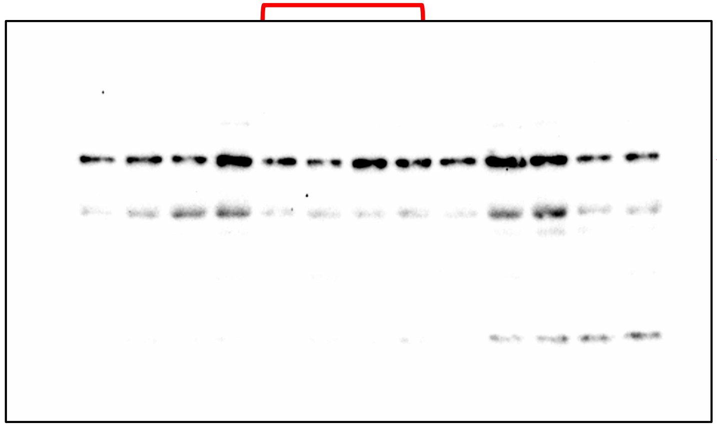

64 kDa

Gr

kDa

250 -
150 -
100 -
75 -
50 -
37 -
25 -
20 -
15 -

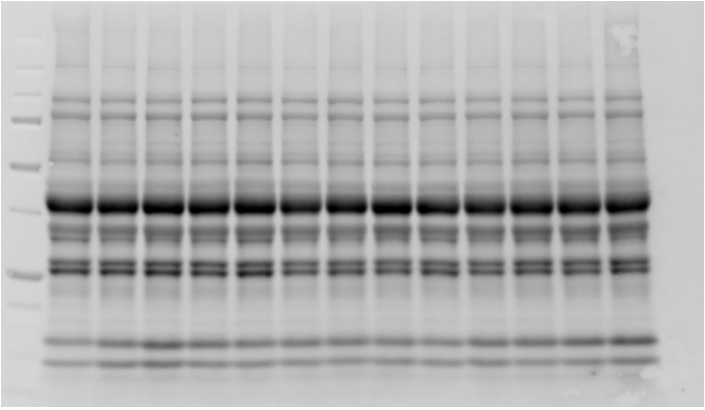

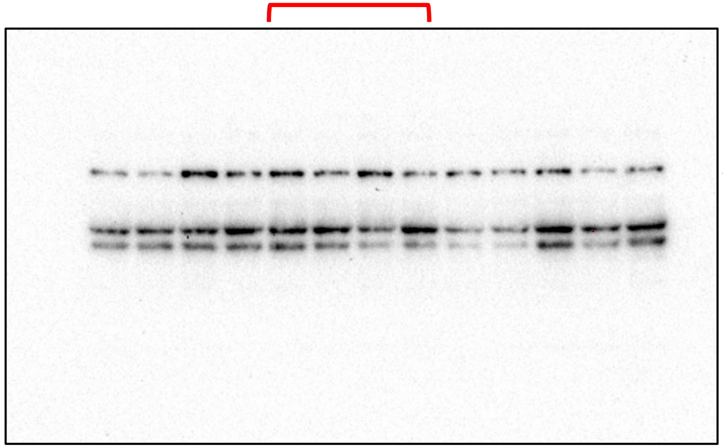

64 kDa

Figure S9

Molecular weight marker: Precision Plus Protein™ All Blue Prestained Protein Standards #1610373

Complete blot images shown in Figure

The lanes used in the figure

CAT

Total protein images after electrophoresis followed by trans-blot to membrane used as loading control

Specific bands of target protein detected after immunostaining

Gw

kDa
250 -
150 -
100 -
75 -
50 -
37 -
25 -
20 -
15 -

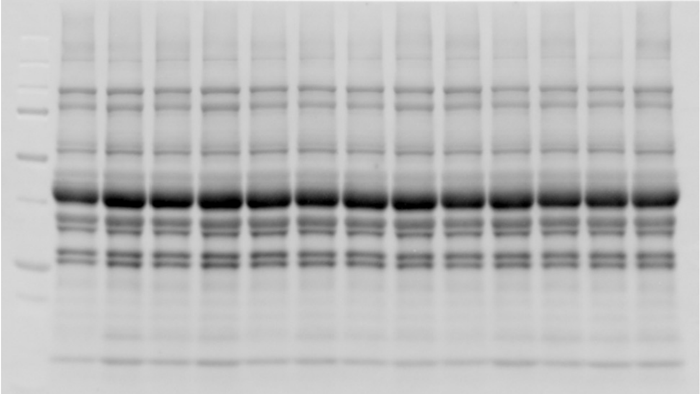

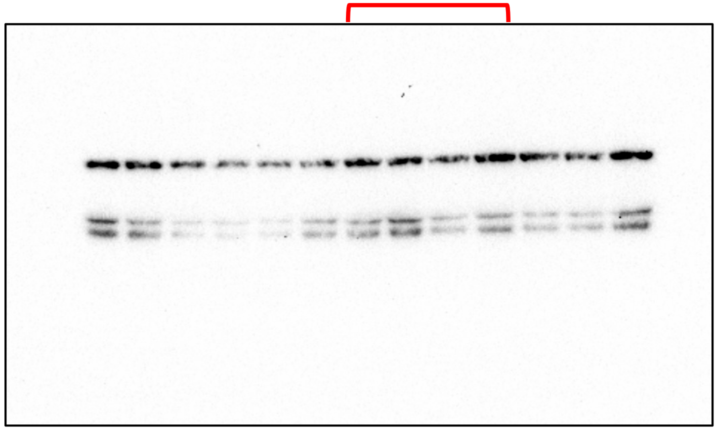

55 kDa

PL

kDa
250 -
150 -
100 -
75 -
50 -
37 -
25 -
20 -
15 -

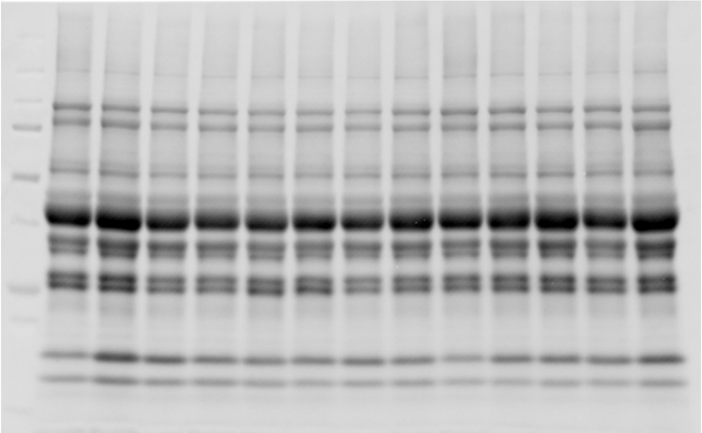

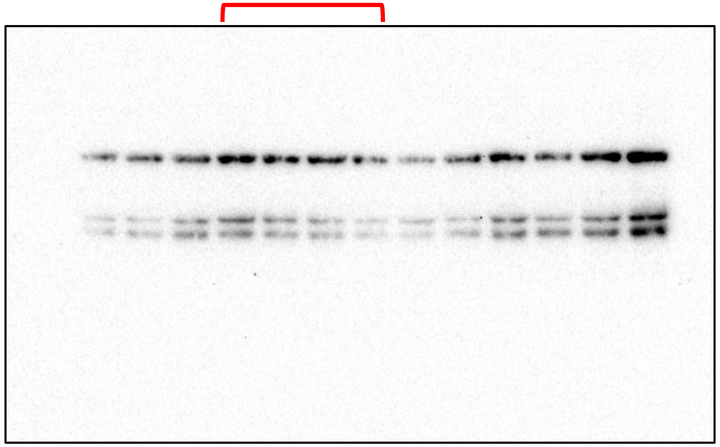

55 kDa

Figure S10

Molecular weight marker: Precision Plus Protein™ All Blue Prestained Protein Standards #1610373

Complete blot images shown in Figure

The lanes used in the figure

CAT

Total protein images after electrophoresis followed by trans-blot to membrane used as loading control

Specific bands of target protein detected after immunostaining

DIA

kDa
250 -
150 -
100 -
75 -
50 -
37 -
25 -
20 -
15 -

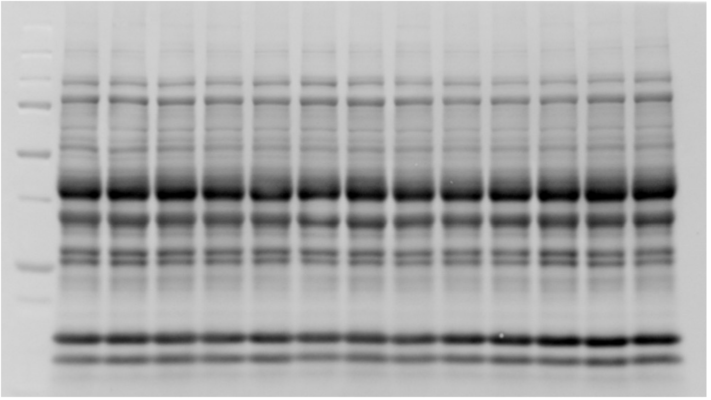

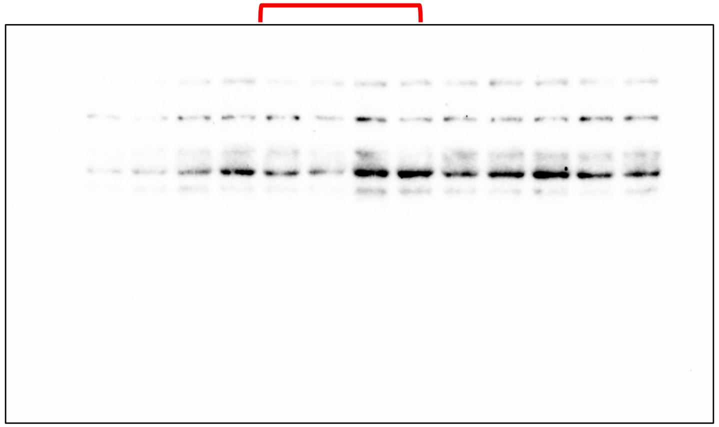

84 kDa
55 kDa

LV

kDa
250 -
150 -
100 -
75 -
50 -
37 -
25 -
20 -
15 -

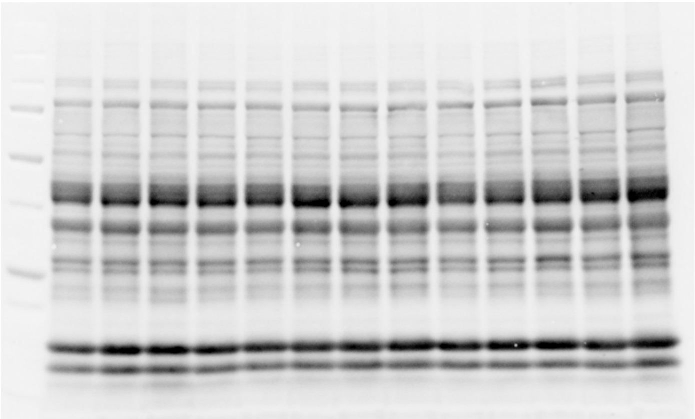

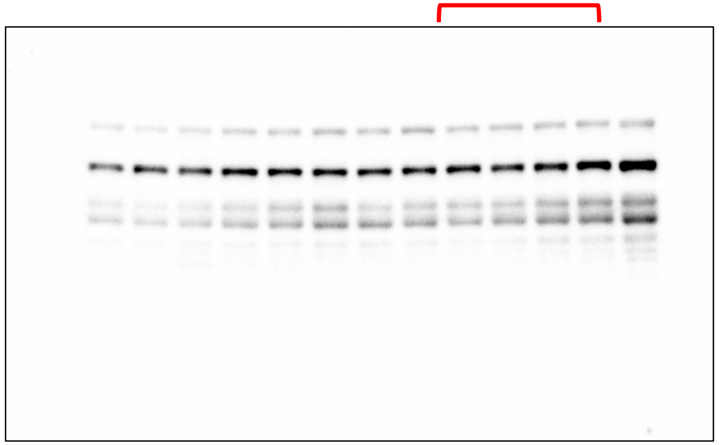

84 kDa
55 kDa

Figure S11

Complete blot images shown in Figure

The lanes used in the figure

GPX1

Molecular weight marker: Precision Plus Protein™ All Blue Prestained Protein Standards #1610373

Total protein images after electrophoresis followed by trans-blot to membrane used as loading control

Specific bands of target protein detected after immunostaining

SOL

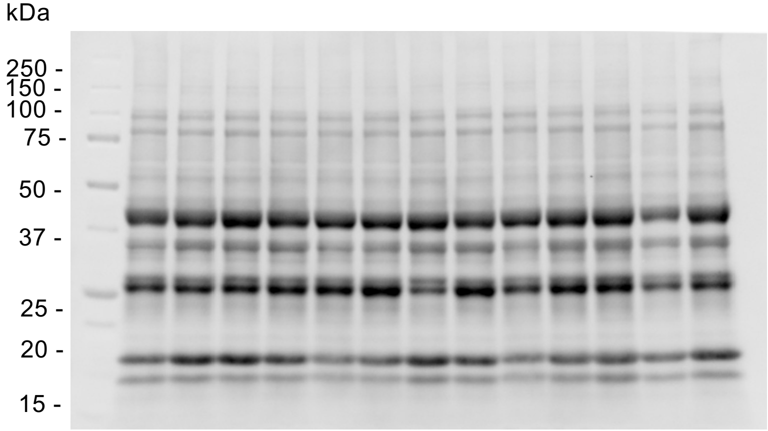

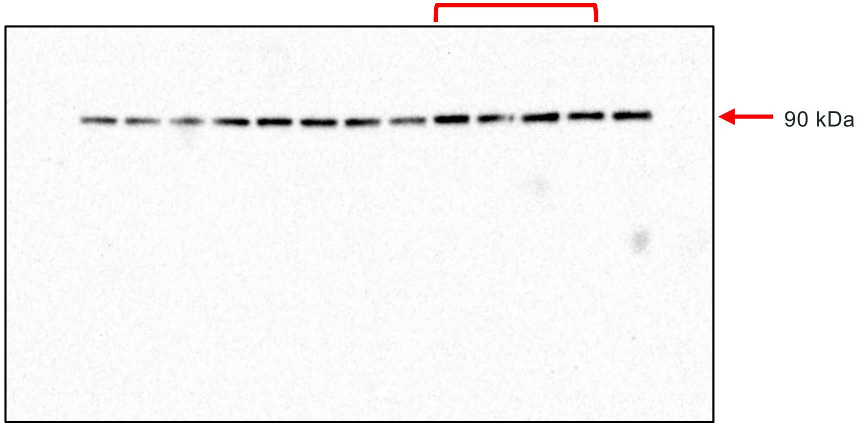

Gr

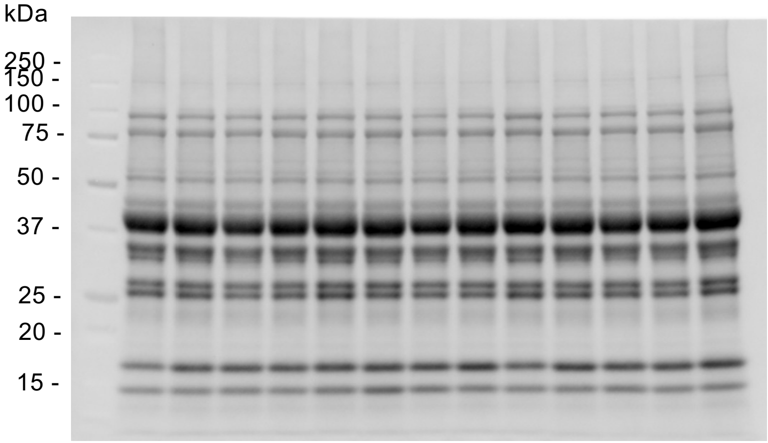

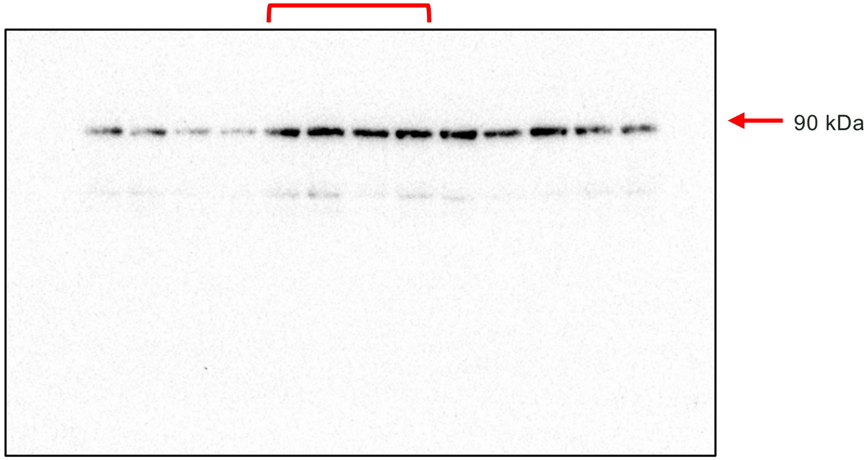

Figure S12

Complete blot images shown in Figure

The lanes used in the figure

Molecular weight marker: Precision Plus Protein™ All Blue Prestained Protein Standards #1610373

GPX1

Total protein images after electrophoresis followed by trans-blot to membrane used as loading control

Specific bands of target protein detected after immunostaining

Gw

kDa
250 -
150 -
100 -
75 -
50 -
37 -
25 -
20 -
15 -

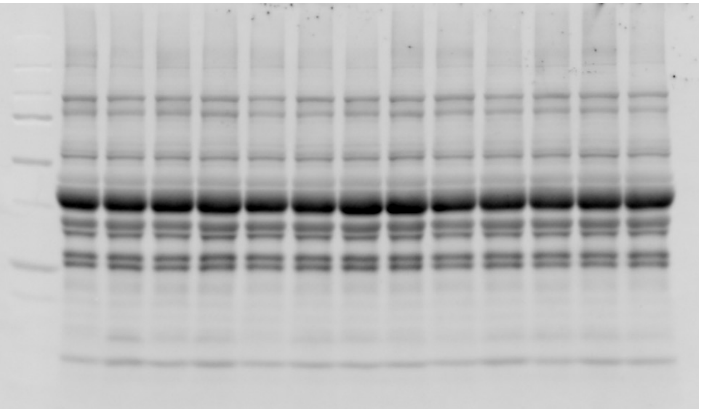

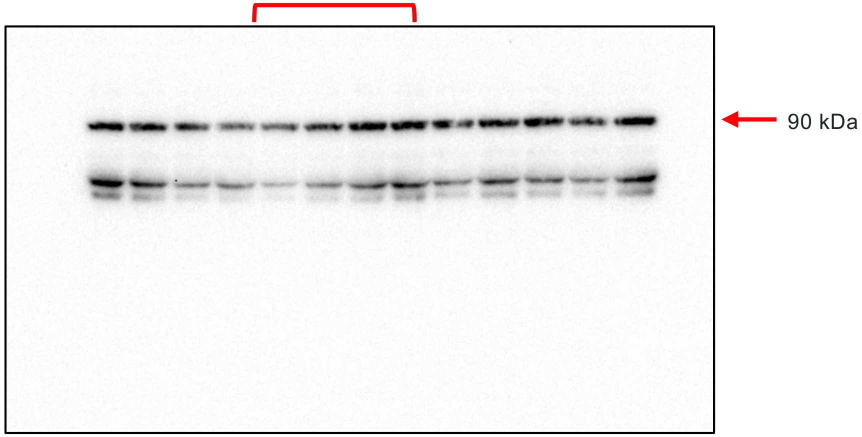

PL

kDa
250 -
150 -
100 -
75 -
50 -
37 -
25 -
20 -
15 -

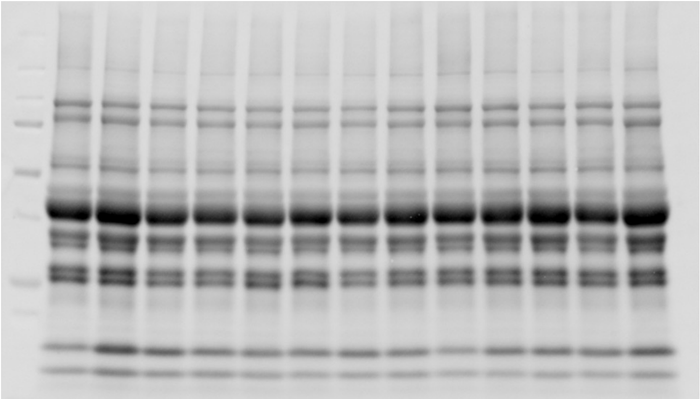

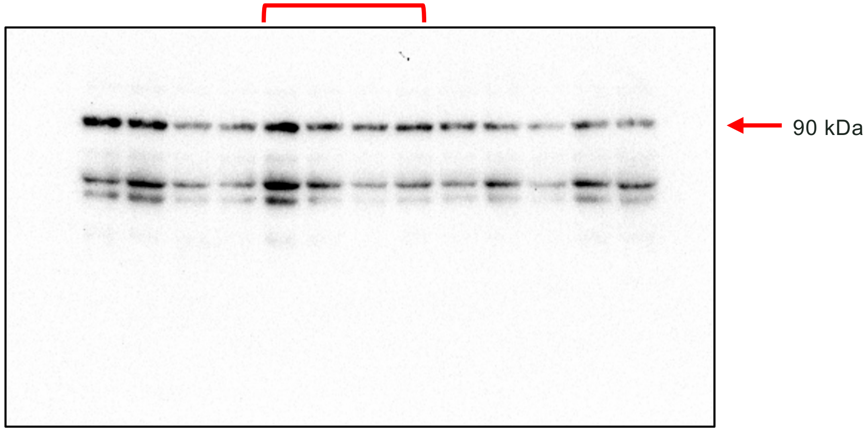

Figure S13

Molecular weight marker: Precision Plus Protein™ All Blue Prestained Protein Standards #1610373

Complete blot images shown in Figure

The lanes used in the figure

GPX1

Total protein images after electrophoresis followed by trans-blot to membrane used as loading control

Specific bands of target protein detected after immunostaining

DIA

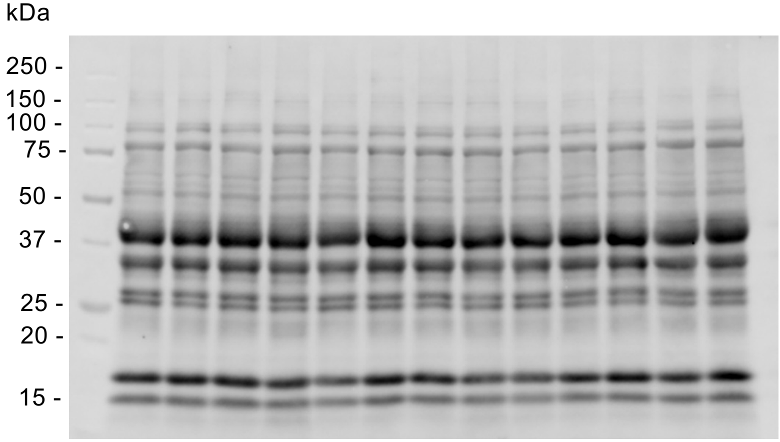

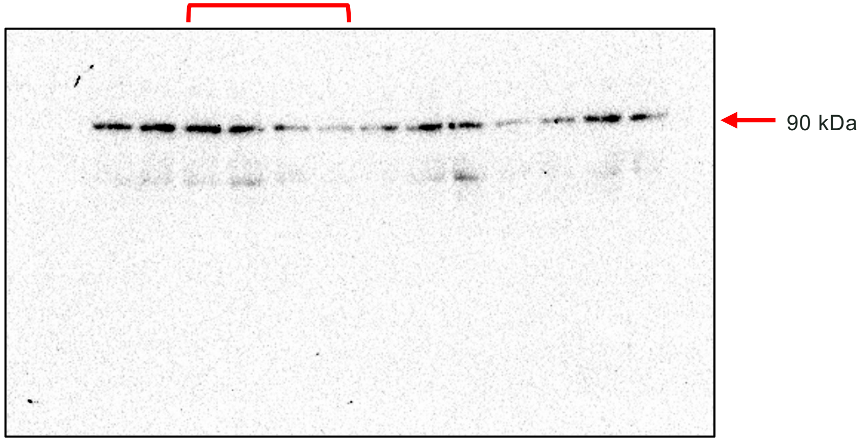

LV

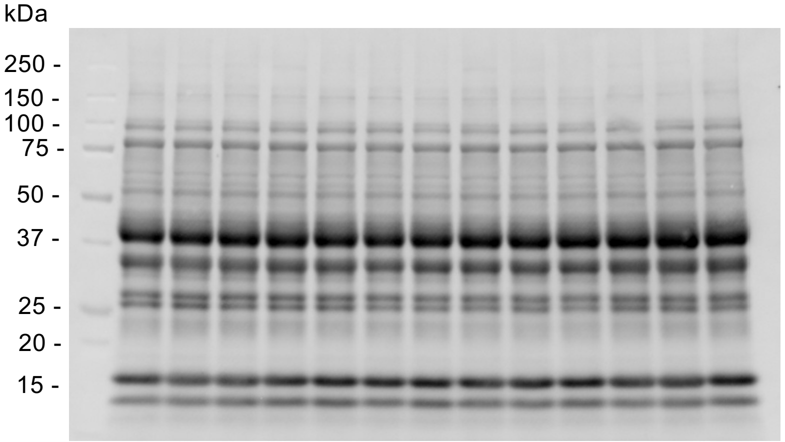

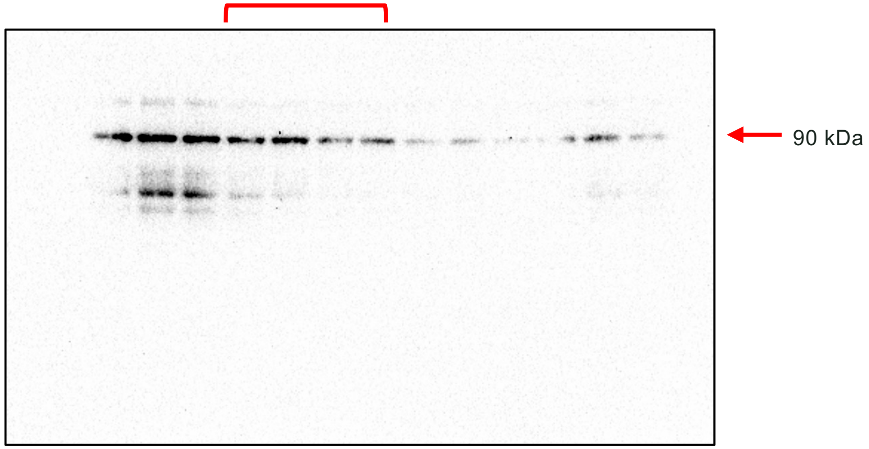

Figure S14

Molecular weight marker: Precision Plus Protein™ All Blue Prestained Protein Standards #1610373

Complete blot images shown in Figure

The lanes used in the figure

PGC1α

Total protein images after electrophoresis followed by trans-blot to membrane used as loading control

Specific bands of target protein detected after immunostaining

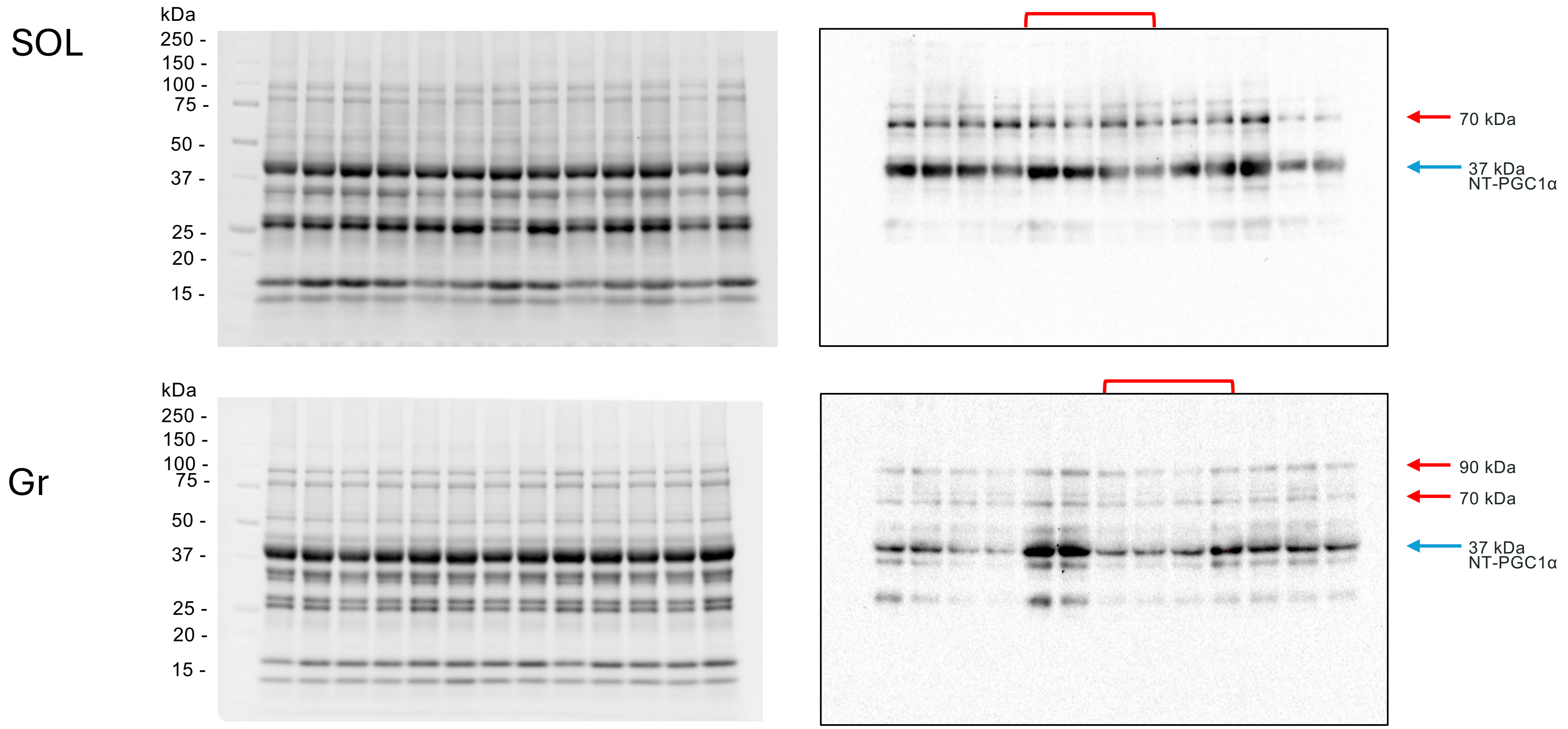

Figure S15

Molecular weight marker: Precision Plus Protein™ All Blue Prestained Protein Standards #1610373

Complete blot images shown in Figure

The lanes used in the figure

PGC1α

Total protein images after electrophoresis followed by trans-blot to membrane used as loading control

Specific bands of target protein detected after immunostaining

Gw

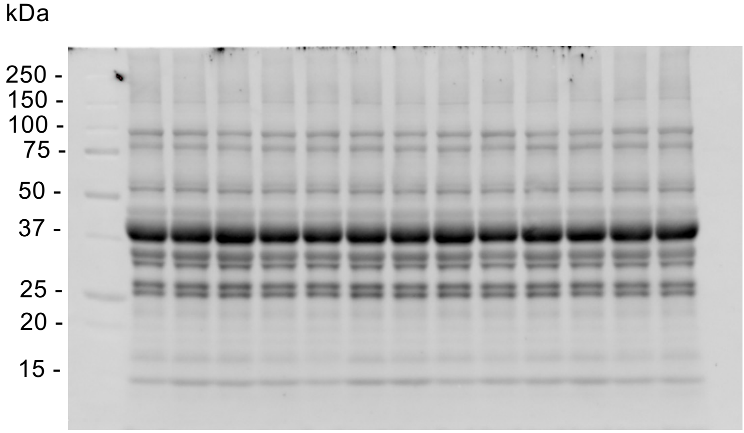

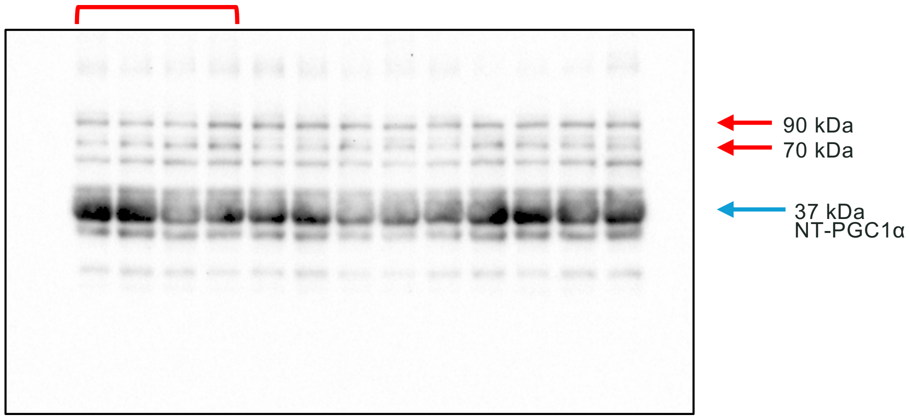

PL

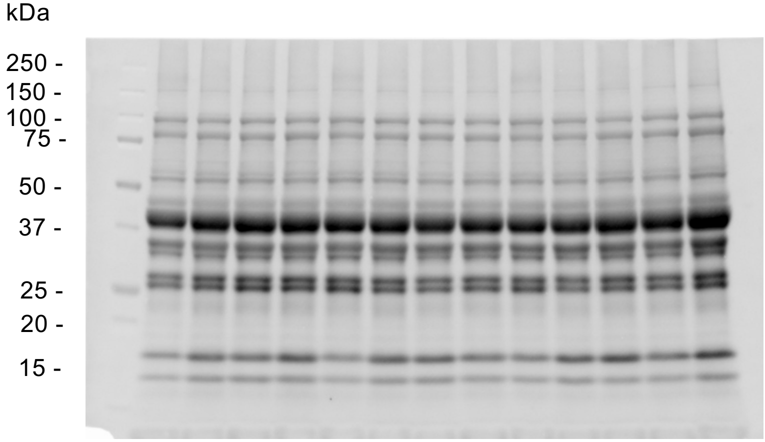

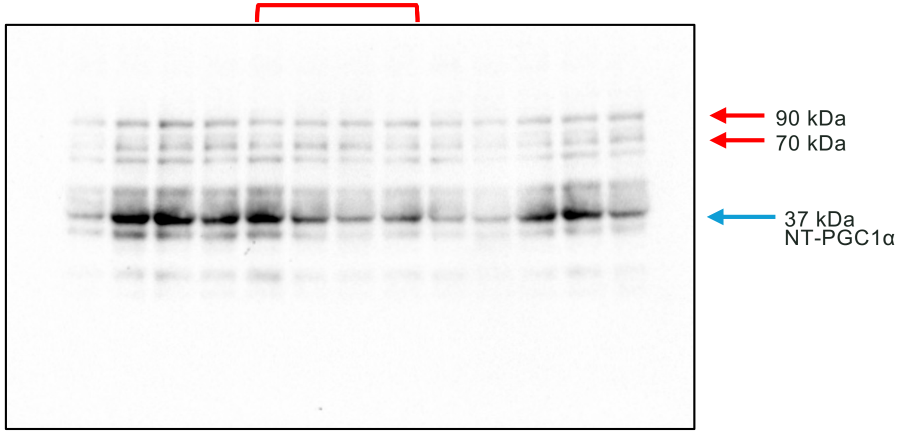

Figure S16

Molecular weight marker: Precision Plus Protein™ All Blue Prestained Protein Standards #1610373

Complete blot images shown in Figure

The lanes used in the figure

PGC1α

Total protein images after electrophoresis followed by trans-blot to membrane used as loading control

Specific bands of target protein detected after immunostaining

DIA

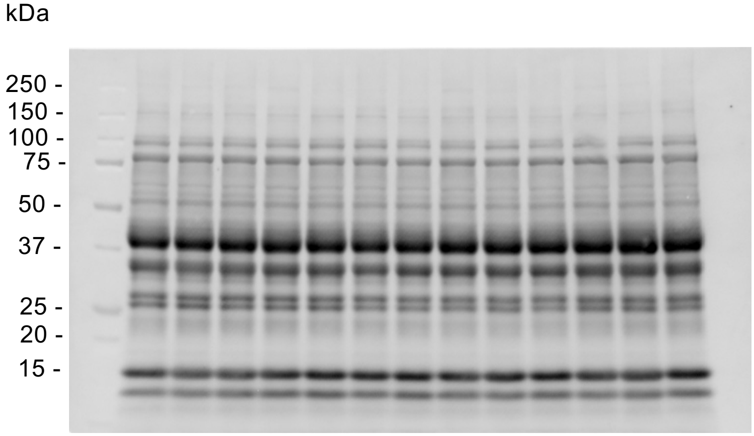

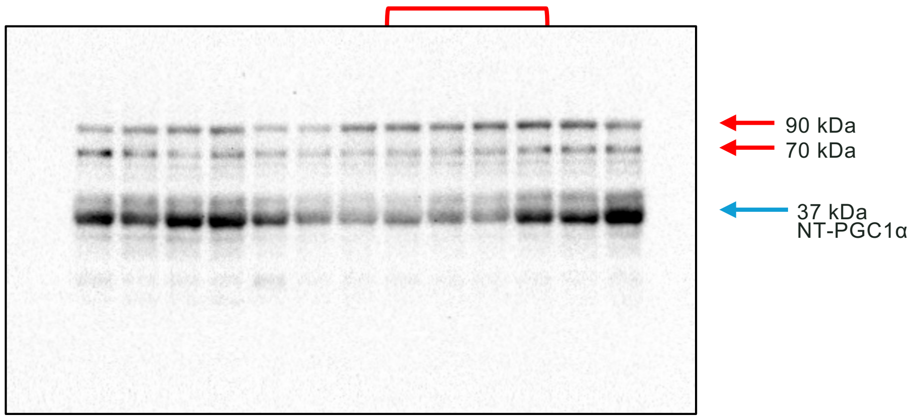

LV

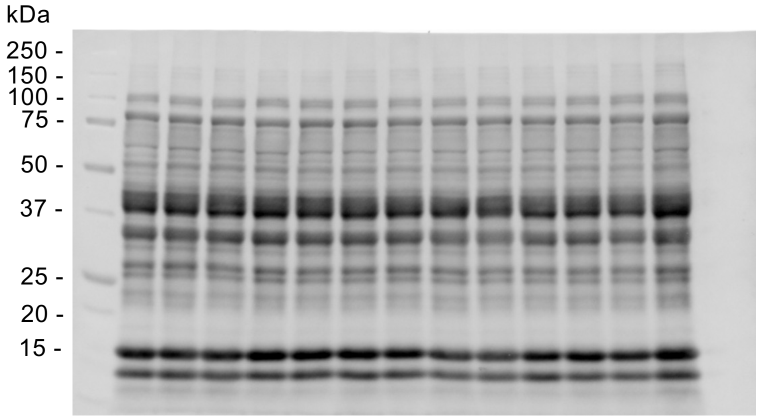

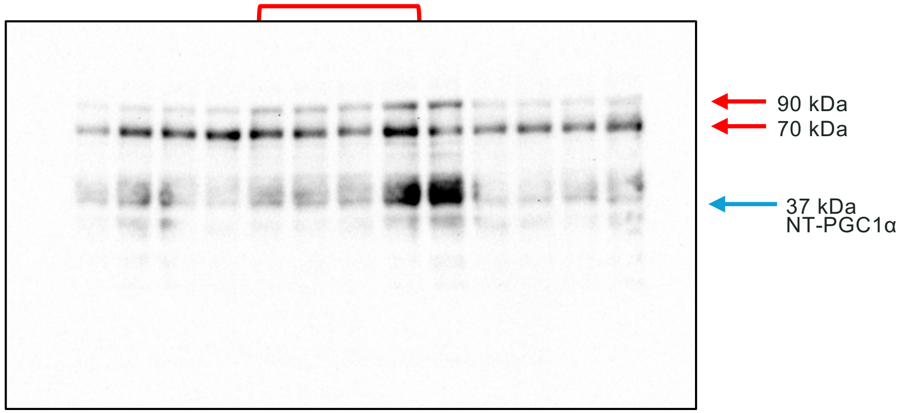

Supplement: Supplementary file 1 — Figures S1–S16. [file PHY2-14-e71020-s001.zip › PHYSREP-2026-05-442-s01.pdf]
