# Supplementary material for: Effects of different oxygen concentrations during intermittent hyperoxic training on endurance capacity in well‐trained male mice
Source: Physiol Rep. 2026 Jul 18;14(14):e71020. doi: 10.14814/phy2.71020 (PMC13379783; doi:10.14814/phy2.71020)
Supplement: Supplementary file 1 — Figures S1–S16. [file PHY2-14-e71020-s001.zip › PHY2_71020__pe.docx]

Caption for supplemental figures. MS# PHY2_71020

**Figure S1.**  Running distance per day during voluntary wheel training

Values are expressed as box and whisker plots with 5th, 25th, 50th, 75th and 95th percentile. Dots in the figure indicate data for each mouse. Bayes factors are in parentheses. φ, The 95% confidential interval did not contain the mean value of target group for comparison.

**Figure S2.**  Graded ramp treadmill running protocol for the endurance capacity test. The values of work (J) in the figure are calculated for a body weight is 40 g.

**Figure S3.** Representative immunofluorescent images of muscle fiber profiles for red portion of gastrocnemius. (a and e), SED; (b and f), ET; (b and f), INT50; (c and g), INT75 (d and h) groups. (a-d), type I fiber; (e-h), type IIA fiber. Horizontal bars represent 20 µm.

**Figure S4.** Representative images for capillary profiles (a-d) and muscle fiber profiles (e-h) of plantaris muscle (GrL) for SED (a and e), ET (b and f), INT50 (c and g), and INT75 (d and h) groups. Horizontal bars represent 20 µm.

**Figure S5.** Western blot images of total protein (left panel) and specific bands of the target protein (right panel). The U-shaped bands are shown in Figure 7a (SOL and Gr).

**Figure S6.** Western blot images of total protein (left panel) and specific bands of the target protein (right panel). The U-shaped bands are shown in Figure 7a (Gw and PL).

**Figure S7.** Western blot images of total protein (left panel) and specific bands of the target protein (right panel). The U-shaped bands are shown in Figure 7a (DIA and LV).

**Figure S8.** Western blot images of total protein (left panel) and specific bands of the target protein (right panel). The U-shaped bands are shown in Figure 7b (SOL and Gr).

**Figure S9.** Western blot images of total protein (left panel) and specific bands of the target protein (right panel). The U-shaped bands are shown in Figure 7b (Gw and PL).

**Figure S10.** Western blot images of total protein (left panel) and specific bands of the target protein (right panel). The U-shaped bands are shown in Figure 7b (DIA and LV).

**Figure S11.** Western blot images of total protein (left panel) and specific bands of the target protein (right panel). The U-shaped bands are shown in Figure 7c (SOL and Gr).

**Figure S12.** Western blot images of total protein (left panel) and specific bands of the target protein (right panel). The U-shaped bands are shown in Figure 7c (Gw and PL).

**Figure S13.** Western blot images of total protein (left panel) and specific bands of the target protein (right panel). The U-shaped bands are shown in Figure 7c (DIA and LV).

**Figure S14.** Western blot images of total protein (left panel) and specific bands of the target protein (right panel). The U-shaped bands are shown in Figure 8 (SOL and Gr).

**Figure S15.** Western blot images of total protein (left panel) and specific bands of the target protein (right panel). The U-shaped bands are shown in Figure 8 (Gw and PL).

**Figure S16.** Western blot images of total protein (left panel) and specific bands of the target protein (right panel). The U-shaped bands are shown in Figure 8 (DIA and LV).
